# Alcohol drinking and gastric cancer risk: a meta-analysis of observational studies

**DOI:** 10.18632/oncotarget.20918

**Published:** 2017-09-15

**Authors:** Peng-Liang Wang, Fang-Tao Xiao, Bao-Cheng Gong, Fu-Nan Liu

**Affiliations:** ^1^ Department of Surgical Oncology, The First Affiliated Hospital of China Medical University, Shenyang, China

**Keywords:** dietary habit, gastric cancer, cancer prevention, epidemiology

## Abstract

**Background:**

Many studies investigated the association between alcohol drinking and gastric cancer risk, but the results were controversial. We performed a meta-analysis of observational studies to explore the association.

**Materials and Methods:**

We searched PubMed to identify the relevant studies that reported the association between alcohol drinking and gastric cancer risk up to December 31, 2016. We pooled relative risks (RRs) in random effects model and performed dose-response analysis to quantify the association. Cochran *Q* test and I^2^ analyses were used to evaluate the heterogeneity. Meta-regression, subgroup, sensitivity and publication bias analyses were also performed.

**Results:**

75 studies were included in our study. The pooled RR of high vs low total alcohol drinking was 1.25 (95% CI, 1.15–1.37, *P* < 0.001), and a nonlinear association was further observed. Subgroup analysis showed that alcohol drinking significantly associated with the risk of gastric noncardia cancer (RR, 1.19; 95% CI, 1.01–1.40, *P* = 0.033), but not with the risk of gastric cardia cancer (RR, 1.16; 95% CI, 0.98–1.39, *P* = 0.087). Notably, the pooled RRs of high vs low analyses were 1.13 (95% CI, 1.03–1.24, *P* = 0.012) for beer drinking, 1.22 (95% CI, 1.06–1.40, *P* = 0.005) for liquor drinking, and 0.99 (95% CI, 0.84–1.16, *P* = 0.857) for wine drinking.

**Conclusions:**

Our meta-analysis found a nonlinear association between alcohol drinking and gastric cancer risk, and heavy drinking level was strongly related to gastric cancer risk. Beer and liquor had significant positive associations with gastric cancer risk, while wine drinking would not increase gastric cancer risk. These results need to be verified in future research.

## INTRODUCTION

Gastric cancer is the fifth most common cancer and the third most common cause of death from cancer worldwide [1]. In recent years, the numbers of new cases and deaths from gastric cancer continue to increase for population growth and ageing. Life style and dietary habits have been investigated substantially for their relationship with the risk of gastric cancer.

Alcohol was classified as the first class carcinogen by IARC [2]. The previous studies indicated that alcohol drinking is a risk factor for cancer of oral cavity pharynx larynx, esophagus, female breast cancer and colorectal, but a protective factor for non-Hodgkin lymphoma and renal cell carcinoma [3].

For gastric cancer, the effect of alcohol in gastric cancer is still in controversial. Some experimental studies administrated that ethanol itself, its metabolites, and carcinogenic substances in food which may penetrate into the gastric mucosa damaged by the stimulation of ethanol, could induce gastric cancer genesis [4]. While other studies showed that ethanol may be protective for the bactericidal effect of ethanol on *Helicobacter pylori* [5], which are most associated positively with gastric cancer [6]. Several previous meta-analyses have also investigated this topic before [7–13] and some of these studies analyzed the dose-response association between alcohol consumption and gastric cancer risk [10–12]. However, the results of linear association or non-linear association between alcohol and gastric cancer risk were different [10, 11, 13]. In addition, some authors further presented that alcoholic beverages are likely to contain substances other than alcohol which had effects on the gastric mucosa [14], and different alcoholic beverages could have different effects on acid secretion, gastrin release, and gastric emptying [15–18]. But, only one recent study analyzed the different effects of different types of alcoholic beverages on gastric cancer risk [13]. However, the dose-risk association of different types of alcoholic beverages is still unknown.

Therefore, to assess comprehensively and precisely the potential association between alcohol drinking and gastric cancer risk, we performed the present meta-analysis with the current case-control and cohort studies included. And our study mainly focused on the dose-response association between alcohol consumption and gastric cancer risk, especially the different types of alcoholic beverages.

## MATERIALS AND METHODS

### Search strategy

Two authors (WPL and LFN) independently searched the published literature in PubMed up to 31st December 2016 for all the relative studies. The medical subject heading (MeSH) terms and the key terms were used as follows: “*gastric cancer*”, “*stomach neoplasm*”, “*alcohol drinking*” and “*alcoholic beverages*”. The search was limited to English language and human studies. We also scanned the reference articles of all relative studies and review articles.

### Eligibility criteria

Studies included need to meet following criteria:1) the study design as case-control, nested case-control or cohort study, 2) the study investigating the relationship between gastric cancer risk and alcohol drinking, 3) the study supplying relative risk estimate [odds ratio (OR), the relative risk (RR) or hazard ratio (HR)] and corresponding 95% confidence intervals (CIs). When several publications reported the same study, we selected the publication with the most complete information. Two authors (WPL and LFN) assessed independently all the articles for potentially eligible studies. We first reviewed all the identified titles and abstracts, and then full-texts for the articles which met the inclusion criteria or for which eligibility was uncertain. Inconsistencies were adjudicated by discussion and consensus.

### Data extraction

For each study, we extracted the characteristics of the studies (e.g. the last name of the first author, publication year, study design, country, numbers of cases, controls or cohort size and follow-up periods for cohort studies), the characteristics of the study populations (e.g. sex distribution, cancer site and type of alcoholic beverages), outcome measurements (i.e. RR, OR, or HR and its corresponding 95% CIs for the highest vs lowest exposure level), and the main confounders. For each study, multivariate-adjusted risk estimates were used whenever available, otherwise, the unadjusted RRs were extracted.

Stratification by drinking amount was used to identify the highest and lowest exposure level if available, otherwise drinking frequency was used. The drinkers and nondrinkers were identified as the highest to lowest intake if the studies only compared the two groups. Alcohol drinking levels were generally defined as ‘light drinker’ (1 drink per drinking day), ‘moderate drinker’ (2 drinks) and ‘heavy drinker’ (3+ drinks). However, the definitions of how many grams of ethanol contained in one drink is different in each country. Thus, estimation of ethanol intake is complicated. In our present study, we used 12.5 grams of ethanol as the standard measurement of one drink as many previous studies described [7, 10, 11, 19].

### Quality assessment

Newcastle-Ottawa Scale was used to perform study quality assessment [20]. The highest score was 9, including 4 for population selection, 2 for comparability, and 3 for exposure or outcomes of population. A score of > 6 was considered as high quality.

Two authors (GBC and XFT) performed the data extraction and quality assessment independently and cross-checked. Disagreements were resolved by team discussion.

### Statistical analysis

The measure of interest was the RR [or the odds ratios (OR) in case-control studies and the hazard ratio (HR) in the cohort studies]. To quantify the association between alcohol drinking and gastric cancer risk, we used a random-effects model to calculate summary relative risks, considering within- and between-study variability [21]. For studies that reported different sexes, races, subtypes of beverages, cancer sites and histological types separately, we obtained overall relative risks for each study by using the method proposed by Hamling [22]. For those which lacked necessary information for applying the Hamling method, the random-effect model was applied.

We performed dose-response meta-analysis using the method provided by Greenland and Longnecker [23] and Orsini [24]. The studies included in dose-response analysis should have no less than 3 quantitative exposure categories with the same reference group, and each category should provide the numbers of cases and person-years or non-cases and the relative estimates with their corresponding 95% CI. The median dose was computed as midpoint of lower and upper boundaries if not reported. The lowest exposure level was considered as zero when it was open-ended, and the highest exposure level was calculated by 1.2 times of its lower bound when open-ended [25]. For studies that reported dose in volume, the dose was calculated using the standard concentration, that is, 100 ml of alcohol, beer, liquor and wine contains about 80 g, 5 g, 40 g, and 15 g of ethanol respectively [2]. The results of dose-response analysis were presented for per 12.5 g/day (about 1 drink/day) increment. We evaluated the potential non-linear relationship using restricted cubic spline model with 3 knots at percentiles 10%, 50% and 90% of the distribution of alcohol drinking. *P* values for nonlinearity were calculated by testing the null hypothesis that the coefficients of the second spline were equal to zero [26].

Heterogeneity among studies was assessed with the Cochran Q (heterogeneity chi-squared) and I-square statistics, with I-square > 50% representing significant heterogeneity [27]. To find the source of heterogeneity, we performed meta-regression with covariables, such as publication year, geography, sample size, quality score, and study design. Subgroup analysis was further performed to evaluate the effects of the variables which had been identified by meta-regression or considered as the source of heterogeneity. Sensitivity analysis was performed to evaluate the effect of individual study and the stability of our results by omitting each study or some studies and summarizing the remaining. Funnel plot and Begg's test [28] were performed to assess publication bias. Significant publication bias was indicated when *P* values were less than .10.

All the analyses were performed with STATA (version 12.0 Stata Corporation, college station, TX). *P*-value < .05 was considered statistically significant.

## RESULTS

### Search results, study characteristics and quality assessment

**Figure 1:**
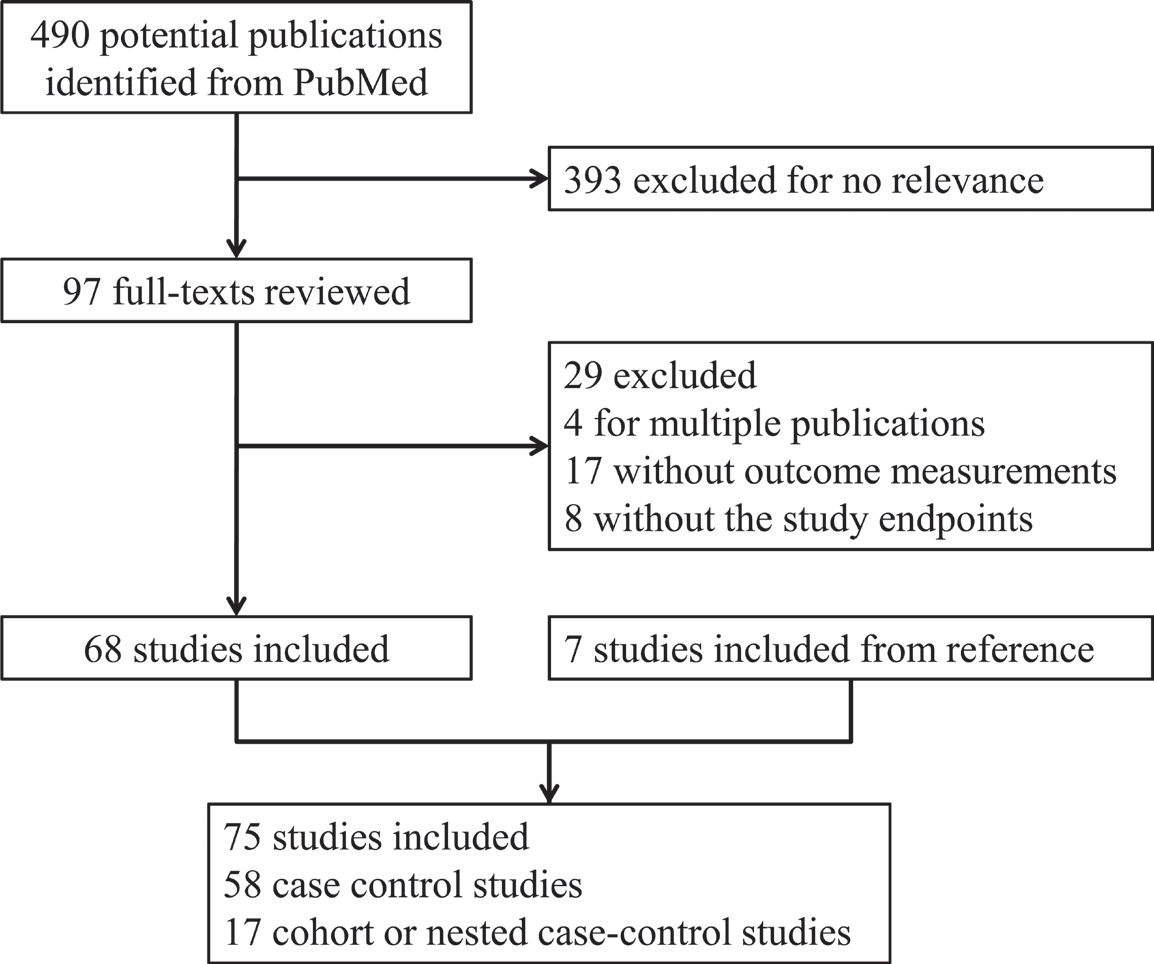
Flow chart of the systematic search of literature on alcohol consumption and the risk of gastric cancer

490 references were generated from PubMed with the search strategy, of which 393 were excluded as non-relevant studies after scanning the titles and abstracts. (Figure 1) For the remaining 97 studies, we retrieved the full texts for detailed evaluation. 29 studies were excluded and reasons were presented in Supplementary material. 7 additional studies were included from the reference review. Finally, 75 studies (58 case-control studies and 17 cohort or nested case-control studies) with 2073591 participants were included in the present meta-analysis. The major characteristics of the included studies are presented in Supplementary Table 1. Of these 75 studies, 73 studies reported total alcohol drinking, 25 studies reported beer drinking, 26 studies reported wine drinking, and 28 studies reported liquor drinking. 22 studies were conducted in Europe, 19 in American, and 34 in Asian.

Quality assessment scores are summarized in Supplementary Table 2. The average scores were 6.48 in total, 6.16for case-control studies, and 7.58 for cohort studies. All of cohort studies were high quality (range 7–9), while less than half of case-control studies (26/58) were high quality (range 4–8).

### Total alcohol

#### High vs low analysis

**Figure 2:**
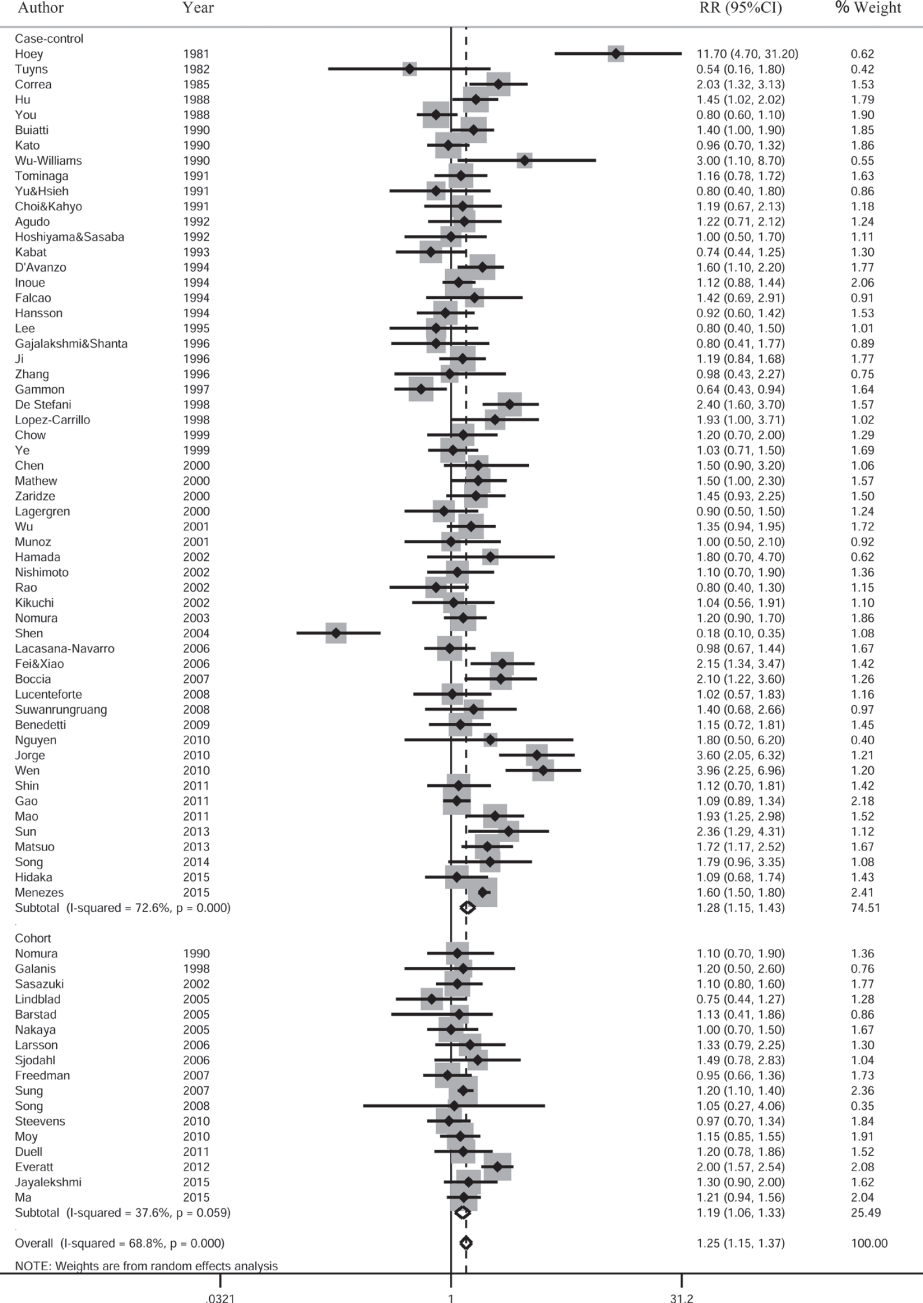
Relatives risk of gastric cancer for the high vs low category of alcohol consumption Studies are grouped according to study design. The pooled RRs were calculated using the random-effects models. Open diamond denote the pooled RR. The size of gray box is positively proportional to the weight assigned to each study (inverse of variance), and horizontal lines represent the 95% confidence intervals. RR, relative risk; CI, confidence interval.

73 studies (56 case-control studies and 17 cohort studies) were included in the pooled analysis of high vs low total alcohol drinking and gastric cancer risk. The pooled RR was 1.25 (95% CI, 1.15–1.37, *P* < 0.001) with significant heterogeneity (I-square = 68.8%, *P* < 0.001) (Figure 2). The funnel plot showed symmetric, and the Begg's test also found no publication bias (*P* = 0.261) (Supplementary Figure 1).

#### Dose-response analysis

**Figure 3:**
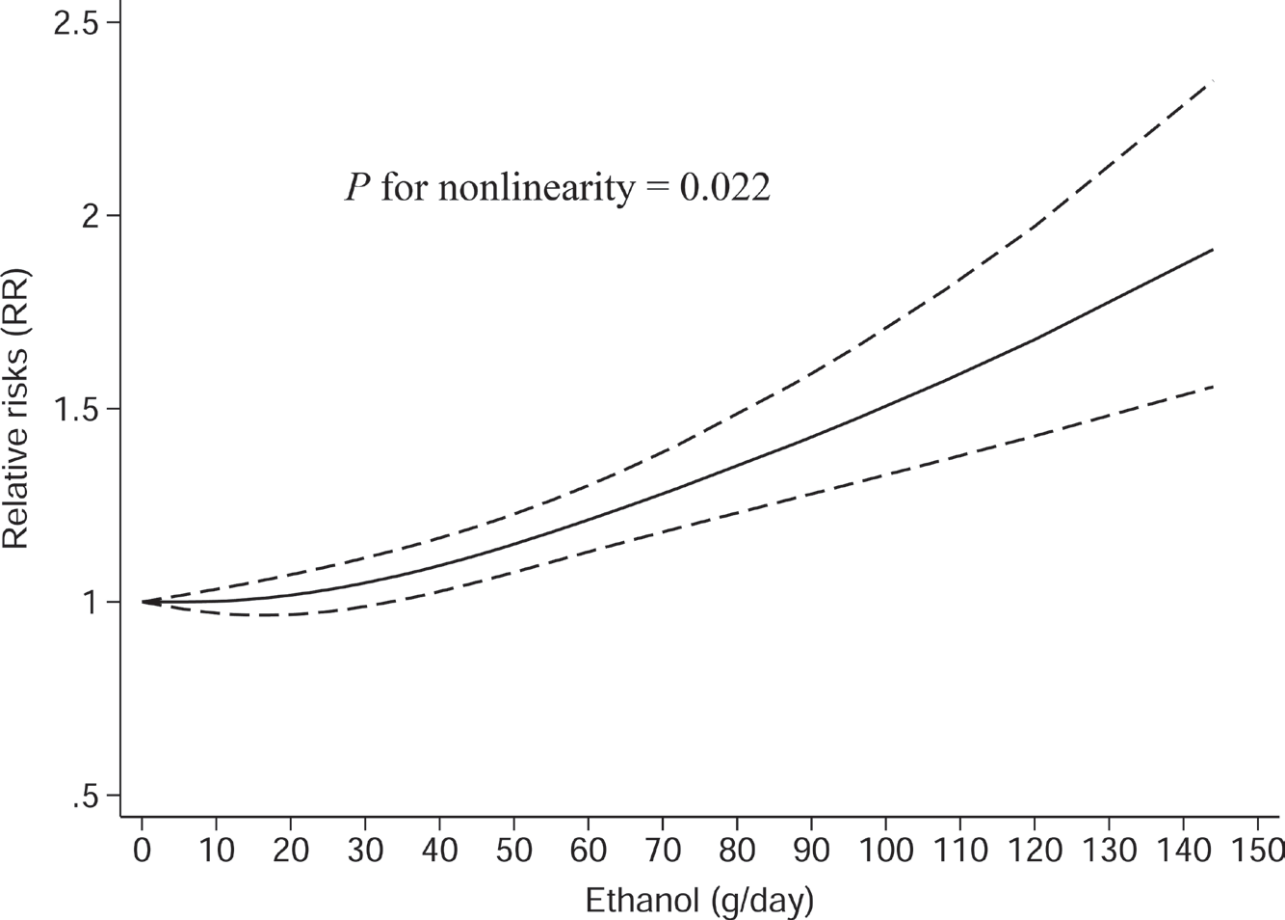
Nonlinear dose-response analysis of the association between total alcohol drinking and gastric cancer risk in studies assessed by restricted cubic spline model with three knots The solid line represented the estimated relative risk and the dashed lines represented the 95% confidence intervals.

28 studies (15 case-control studies and 13 cohort studies) were included in dose-response meta-analysis of total alcohol drinking. The pooled RR for an 12.5 g/day increment of ethanol was 1.04 (95% CI, 1.01–1.07, *P* = 0.005) with significant heterogeneity (I-square = 67.5%, *P* < 0.001) (Supplementary Figure 2). A curvilinear association was observed between total alcohol drinking and gastric cancer risk (P_for nonlinearity_ = 0.022) (Figure 3).

### Beer

#### High vs low analysis

**Figure 4:**
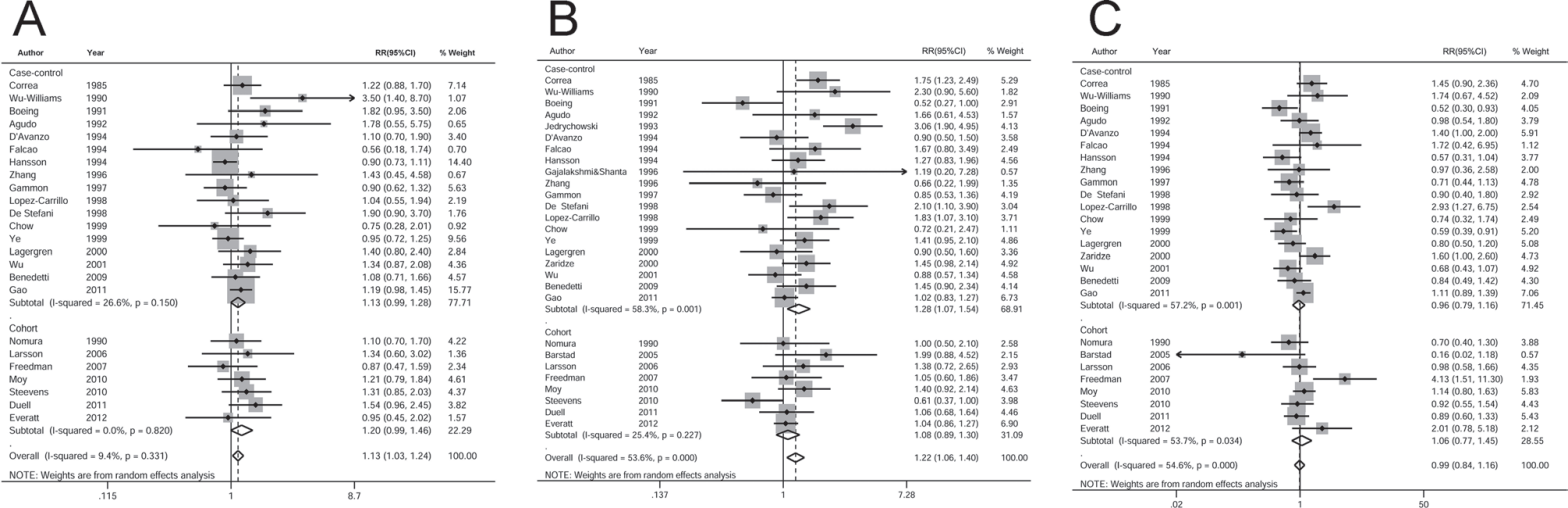
Relatives risk of gastric cancer for the high vs low categories of (**A**) beer, (**B**) liquor, and (**C**) wine drinking. The pooled RRs were calculated using the random-effects models. Open diamond denote the pooled RR. The size of gray box is positively proportional to the weight assigned to each study (inverse of variance), and horizontal lines represent the 95% confidence intervals. RR, relative risk; CI, confidence interval.

24 studies (17 case-control studies and 7 cohort studies) were included in the analysis of high vs low beer drinking and gastric cancer risk. The pooled RR was 1.13 (95% CI, 1.03–1.24, *P* = 0.012) without significant heterogeneity (I-square = 9.4%, *P* = 0.331) (Figure 4A).

#### Dose-response analysis

**Figure 5:**
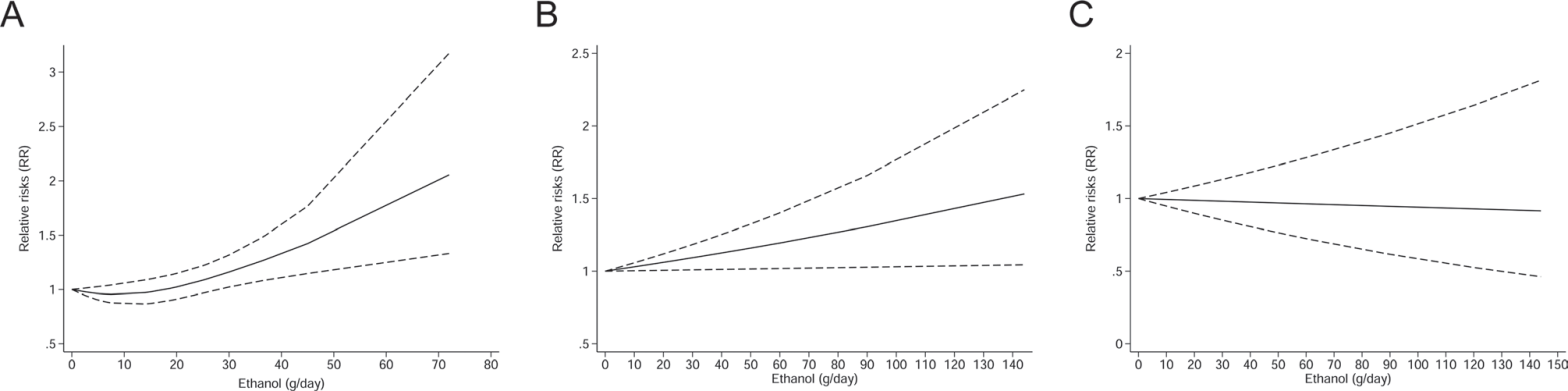
Dose-response analysis of the association between (**A**) beer, (**B**) liquor, and (**C**) wine drinking and gastric cancer risk in studies assessed by restricted cubic spline model with three knots. The solid line represented the estimated relative risk and the dashed lines represented the 95% confidence intervals.

14 studies (9 case-control and 5 cohort studies) were included in the dose-response meta-analysis. The pooled RR for an 12.5 g/day increment of ethanol intake was 1.07 (95% CI, 1.01–1.13, *P* = 0.025) without significant heterogeneity (I-square = 9.5%, *P* = 0.348) (Supplementary Figure 3A). There was evidence of a potential nonlinear association between beer drinking and gastric cancer risk (P_for nonlinearity_ = 0.035) (Figure 5A).

### Liquor

#### High vs low analysis

28 studies (20 case-control studies and 8 cohort studies) were included in the analysis of high vs low liquor drinking and gastric cancer risk. The pooled RR was 1.22 (95% CI, 1.06–1.40, *P* = 0.005) with significant heterogeneity (I-square = 53.6%, *P* < 0.001) (Figure 4B).

#### Dose-response analysis

14 studies (9 case-control and 5 cohort studies) were included in the dose-response meta-analysis. The pooled RR for an 12.5 g/day increment of ethanol was 1.03 (95% CI, 0.98–1.09, *P* = 0.296) without significant heterogeneity (I-square = 24.1%, *P* = 0.193) (Supplementary Figure 3B). There was evidence of a linear association between liquor drinking and gastric cancer risk (P_for nonlinearity_ = 0.269) (Figure 5B)

### Wine

#### High vs low analysis

26 studies (18 case-control studies and 8 cohort studies) were included in the analysis of high vs low wine drinking and gastric cancer risk. The pooled RR was 0.99 (95% CI, 0.84–1.16, *P* = 0.857) with significant heterogeneity(I-square = 54.6%, *P* < 0.001) (Figure 4C).

#### Dose-response analysis

13 studies (9 case-control and 4 cohort studies) were included in the dose-response meta-analysis. The pooled RR for an 12.5 g/day increment of ethanol was 0.99 (95% CI, 0.93–1.06, *P* = 0.769) with significant heterogeneity (I-square = 61.9%, *P* = 0.002) (Supplementary Figure 3C). A linear association between wine drinking and gastric cancer risk was observed. (P_for nonlinearity_ = 0.242) (Figure 5C)

### Subgroup analysis, Meta-regression analysis

To find the source of the heterogeneity, we performed meta-regression and subgroup analyses for total alcohol drinking (Table 1). No variable related to the source of heterogeneity was found in univariate and multivariate meta-regression analyses.

**Table 1:** Subgroup analyses of total alcohol drinking and gastric cancer risk, high vs low intake

	Studies *n*	RR (95% CI)	*P*	I-square (%)	P_heterogeneity_	P_difference_
All study	73	1.25 (1.15, 1.37)	< 0.001	68.8	< 0.001	
Study Design						
Case-control	56	1.28 (1.15, 1.43)	< 0.001	72.6	<0.001	0.450
Cohort	17	1.19 (1.06, 1.34)	0.003	37.6	0. 059	
Geography						
Non-Asian	39	1.31 (1.16, 1.48)	< 0.001	68.3	< 0.001	0.368
Asian	34	1.19 (1.06, 1.34)	0.003	64.7	< 0.001	
Publication year						
≥ 2000	44	1.29 (1.16, 1.43)	< 0.001	70.1	< 0.001	0.534
< 2000	29	1.20 (1.03, 1.39)	0.016	64.4	< 0.001	
Number of cases						
≥ 250	37	1.21 (1.10, 1.34)	< 0.001	67.0	< 0.001	0.478
< 250	36	1.31 (1.10, 1.56)	0.002	70.8	< 0.001	
Study Quality						
≥ 7 Scores	42	1.16 (1.08, 1.24)	< 0.001	24.2	0.083	0.020
< 7 Scores	31	1.47 (1.22, 1.76)	< 0.001	79.1	< 0.001	
Cancer site						
Cardia	15	1.16(0.98, 1.39)	0.087	15.5	0.280	0.841
Non-cardia	18	1.19(1.01, 1.40)	0.033	63.8	< 0.001	
Sex						
Male	34	1.21 (1.06, 1.37)	0.004	68.2	< 0.001	0.945
Female	12	1.18 (0.95, 1.47)	0.138	26.2	0.187	
Cigarette smoking						
Non-smoker	12	1.41 (1.07, 1.85)	0.014	43.2	0.055	0.921
Smoker	8	1.39 (1.10, 1.76)	0.005	47.1	0.067	
H. pylori infection						0.885
Negative	3	1.66 (1.13, 2.43)	0.009	0	0.432	
Positive	3	1.66 (0.87, 3.14)	0.122	75.9	0.016	
Adjusted Confounders						
BMI						
Yes	16	1.14 (0.98, 1.32)	0.095	63.2	< 0.001	0.265
No	57	1.30 (1.17, 1.44)	< 0.001	69.3	< 0.001	
Smoking & Cigarette						
Yes	41	1.21 (1.10, 1.33)	< 0.001	60.1	< 0.001	0.349
No	32	1.33 (1.13, 1.57)	0.001	76.1	< 0.001	
Fruit & Vegetable						
Yes	18	1.22 (1.06, 1.41)	0.005	45.2	0.020	0.807
No	55	1.26 (1.14, 1.40)	< 0.001	72.6	< 0.001	
SES & income						
Yes	18	1.14 (0.96, 1.36)	0.128	55.2	0.003	0.333
No	55	1.29 (1.17, 1.42)	< 0.001	70.9	< 0.001	

BMI: body mass index, SES: socioeconomic status.

**Figure 6:**
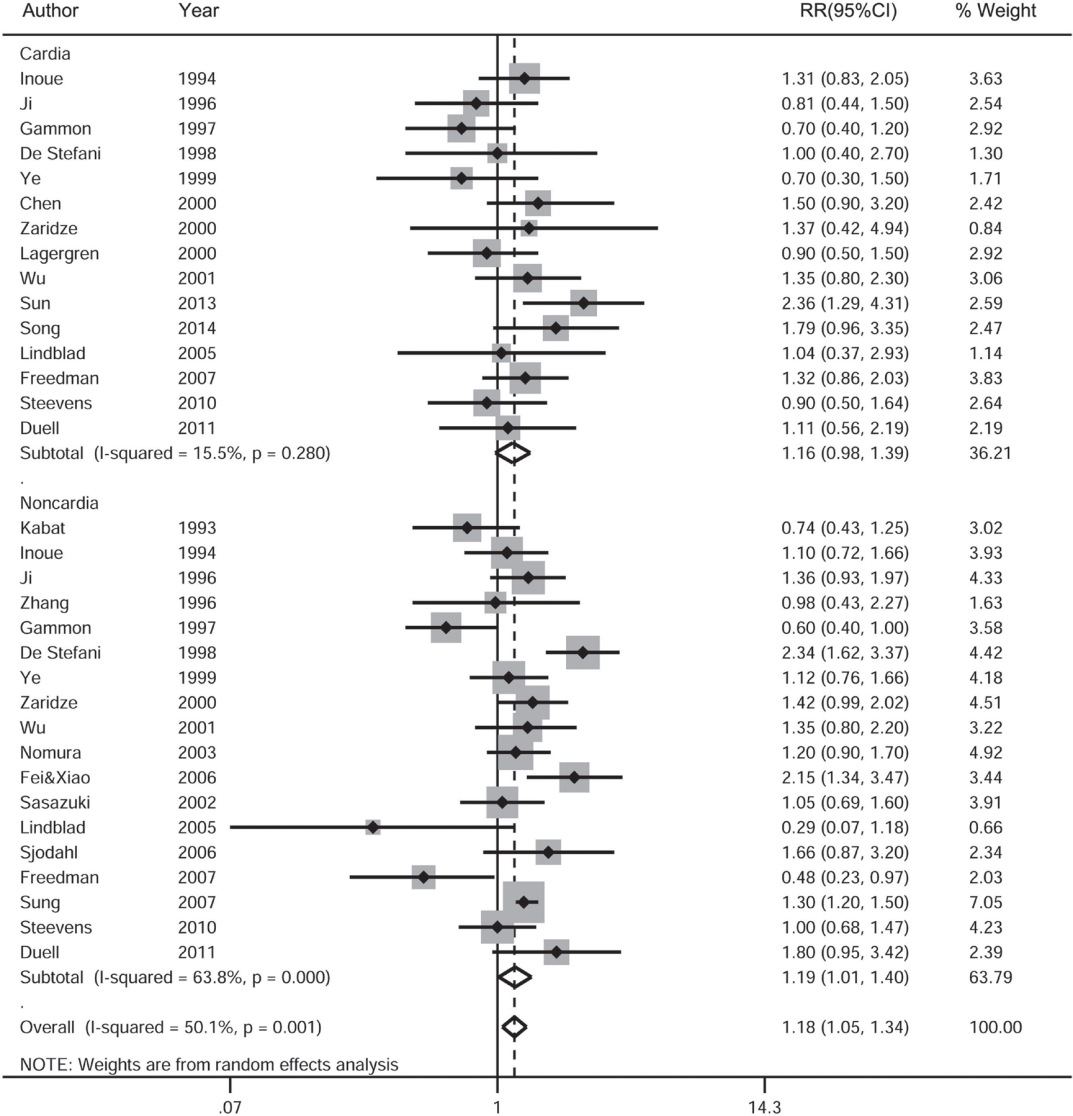
Subgroup analysis of the association between total alcohol drinking and gastric cancer risk based on cancer sites The pooled RRs were calculated using the random-effects models. Open diamond denote the pooled RR. The size of gray box is positively proportional to the weight assigned to each study (inverse of variance), and horizontal lines represent the 95% confidence intervals. RR, relative risk; CI, confidence interval.

Subgroup analyses yielded consistent results, indicating the positive association between total alcohol drinking and gastric cancer risk in all strata, although some of them became statistically insignificant due to small sample size. The subgroup analysis stratified by cancer sites showed that total alcohol drinking had a significant association with the risk of gastric noncardia cancer (RR, 1.19; 95% CI, 1.01–1.40, *P* = 0.033; *n* = 18 studies), but not with the risk of gastric cardia cancer (RR, 1.16; 95% CI, 0.98–1.39, *P* = 0.087; *n* = 15 studies) (Figure 6, Table 1). In the dose-response analysis, the pooled RR of gastric cardia cancer was 1.00 (95% CI, 0.97–1.03, *P* = 0.994; *n* = 7 studies) for an increment of 12.5g/d of ethanol intake, and the pooled RR of gastric noncardia cancer was 1.02 (95% CI, 0.97–1.07, *P* = 0.491; *n* = 8 studies) (Supplementary Figure 4).

### Sensitivity analysis

Sensitivity analysis further approved the stability of the pooled results by omitting one study at a time. The pooled RRs of the remaining studies were stable, with a range from 1.24 (95% CI, 1.13–1.34) to 1.28 (95% CI, 1.18–1.38).

Considering the effects of the excluded studies from dose-response analysis for insufficient data, we repeated the high vs low total alcohol intake analysis restricted to the studies included in dose-response analysis. The pooled RR was 1.20 (95% CI, 1.06–1.36, *P* = 0.004) with significant heterogeneity (I-square = 62.5%, *P* < 0.001), which yielded to consistent results of all the studies included.

## DISCUSSION

In the present meta-analysis with 75 studies and 2073591 participants included, high vs low analysis of total alcohol drinking showed that total alcohol drinking was associated with gastric cancer risk significantly. The dose-response analysis indicated that daily increase of 12.5 g of ethanol (1drink/day) was associated with a 4% increased risk of gastric cancer. A nonlinear association was further found between total alcohol drinking and gastric cancer risk, and the risk increased quickly in heavy alcohol drinking level. Furthermore, alcohol drinking had a significant positive association with gastric noncardia cancer, but not with gastric cardia cancer. Different categories of alcoholic beverages had different effects on gastric cancer risk, that is, beer and liquor drinking had significant positive association with gastric cancer risk, whereas wine drinking had not increased gastric cancer risk.

The present meta-analysis showed that heavy alcohol drinking may increase the risk of gastric cancer, instead of light and moderate alcohol drinking. Based on the previous studies, we inferred that ethanol may be a two-side sword. On the one hand, ethanol and its metabolites, especially acetaldehyde, have carcinogenic and mutagenic effects by modifying DNA via generation of DNA adducts, and inducing oxidative stress and functional genetic variants of alcohol-metabolizing enzymes [29]. Ethanol could also damage gastric mucosa directly [14], which makes carcinogenic substances in food convenient to penetrate into gastric mucosa and induce tumor genesis. On the other hand, ethanol may be protective for the bactericidal effect of ethanol on *H pylori* [5]. Light and moderate alcohol drinking may imply a balance of advantages and disadvantages, or even more advantages, while heavy alcohol drinking may always exhibit the overweighed disadvantages. Besides, heavy alcohol drinking is always associated with malnutrition [30], which may lead to decreased intake of fruit, vegetable and some other compounds with cancer-preventive effect [31]. It should be noted that nondrinkers were not the group with the lowest risk of gastric cancer. A possible explanation is that nondrinkers might be formerly heavy drinkers and had quit due to some disease that could increase gastric cancer risk [32–34].

Most notably, our meta-analysis demonstrated that different categories of alcohol drinking had different effects on gastric cancer risk. Beer and liquor drinking had a significant positive association with gastric cancer risk, whereas wine drinking could not increase gastric cancer risk. In the dose-response meta-analyses, beer consumption showed a nonlinear positive association with gastric cancer risk, while liquor showed a linear association with cancer risk, even though the positive trend was not significant in statistically. Compared with beer and wine, the ethanol concentration of liquor was much higher. Besides, non-alcoholic components in alcohol drinking may also play important roles on gastric cancer risk, for example, nitrosamine (mainly N-nitrosodimethylamine, NDMA) in beer was considered as carcinogenic for gastric cancer [35, 36], whereas polyphenols (mainly resveratrol) rich in wine drinking have the antioxidant [37], anticarcenogenic [38], and ant-inflammatory [39] properties. In addition, wine was considered to have a stronger bactericidal activity than ethanol at the same concentration in a *vitro* study [40]. The beneficial effects of wine drinking may be attributed to all of its components instead of a specific action of one. However, the mechanisms of the different roles of different categories of alcohol drinking on gastric cancer risk need to be further investigated in the future research.

Gastric cancer can be classified as cardia and noncardia subtypes according to the anatomic site. Some authors have investigated that the two subtypes are quite different in single-nucleotide polymorphism [41], causative factors [42] and risk factors [43]. In the present meta-analysis, we observed the positive association between alcohol drinking and the risk of gastric noncardia cancer instead of gastric cardia cancer, which strengthened their differences.

To date, the present meta-analysis provides the most complete and recent evidence on the association between alcohol drinking and gastric cancer risk. First, we included as many related studies as possible, 75 studies with more than2 million of participants. The substantial included population strengthened the reliability of the results. Second, our meta-analysis may be the first to explore the dose-response association of different types of alcohol drinking on gastric cancer risk, and concluded that beer and liquor could increase gastric cancer risk, but wine would not. Third, we performed more relevant methods to improve the accuracy of the results, for example, both linear and non-linear dose-response analyses were applied to quantitate the association between alcohol drinking and gastric cancer risk.

However, there were several limitations in our meta-analysis. First, as the included studies were all observational, the residual confounders are inevitable, such as cigarette smoking, lacking of fruit or vegetable consumption and *H pylori* infection. Although we applied the adjusted measurements whenever available, the confounding effects could not be excluded completely. Majority of included studies were case-control, which may induce more recall and selection biases.

Second, for the absence of sufficient data, some studies were excluded from the dose-response analysis, which may weaken the strength of the conclusions [44]. However, to evaluate the effects of the excluded studies, we repeated high vs low total alcohol intake analysis restricted to the studies included in dose-response analysis, which yielded to consistent results of all studies included.

Third, we applied the uniform standard of 1 drink as 12.5 g of ethanol and the uniform concentration of each type of beverage to calculate the doses, which may cause measurement errors to some extent.

Fourth, significant heterogeneity was observed in high vs low analysis of total alcohol drinking. To find the source of heterogeneity, we performed meta-regression analysis, but no covariate was significantly associated with the heterogeneity in both univariate and multivariate meta-regression analyses. Sensitivity analysis also verified the stability of the pooled results by omitting one study at a time.

Fifth, the subgroup analysis showed significant difference between high and low quality studies (*P* for difference = 0.020), and the association was strengthened by studies with low quality scores. This indicated that our results may be exaggerated in some degree, but it should be noted that the pooled risk estimates of high-quality studies yielded similar results to the original analysis.

### Implication

Gastric cancer is a most common cause of death from cancer worldwide. The association between alcohol drinking and gastric cancer risk remains uncertain, especially different roles of different categories of alcohol drinking. According to the present meta-analysis, daily increase of 12.5 g of ethanol (1 drink/day) was associated with a 4% increased risk of gastric cancer. Heavy alcohol drinking level, beer and liquor drinking were associated with gastric cancer risk significantly, whereas light, moderate, and wine drinking would not increase gastric cancer risk.

## CONCLUSIONS

In conclusion, our meta-analysis found a nonlinear positive association between alcohol drinking and gastric cancer risk, and the risk increased quickly in heavy alcohol drinking level. Alcohol drinking could increase the risk of gastric noncardia cancer significantly, instead of gastric cardia cancer. Beer and liquor had significant positive associations with gastric cancer risk, while wine drinking would not increase gastric cancer risk. However, we should explain the results with cautions for all the limitations, and further research would be needed to confirm our conclusions.

## SUPPLEMENTARY MATERIALS FIGURES AND TABLES






