# Supplementary material for: Alcohol drinking and gastric cancer risk: a meta-analysis of observational studies
Source: Oncotarget. 2017 Sep 15;8(58):99013–23. doi: 10.18632/oncotarget.20918 (PMC5716786; doi:10.18632/oncotarget.20918)
Supplement: Supplementary file 1 [file oncotarget-08-99013-s001.pdf]

Alcohol drinking and gastric cancer risk: a meta-analysis of observational studies

SUPPLEMENTARY MATERIALS

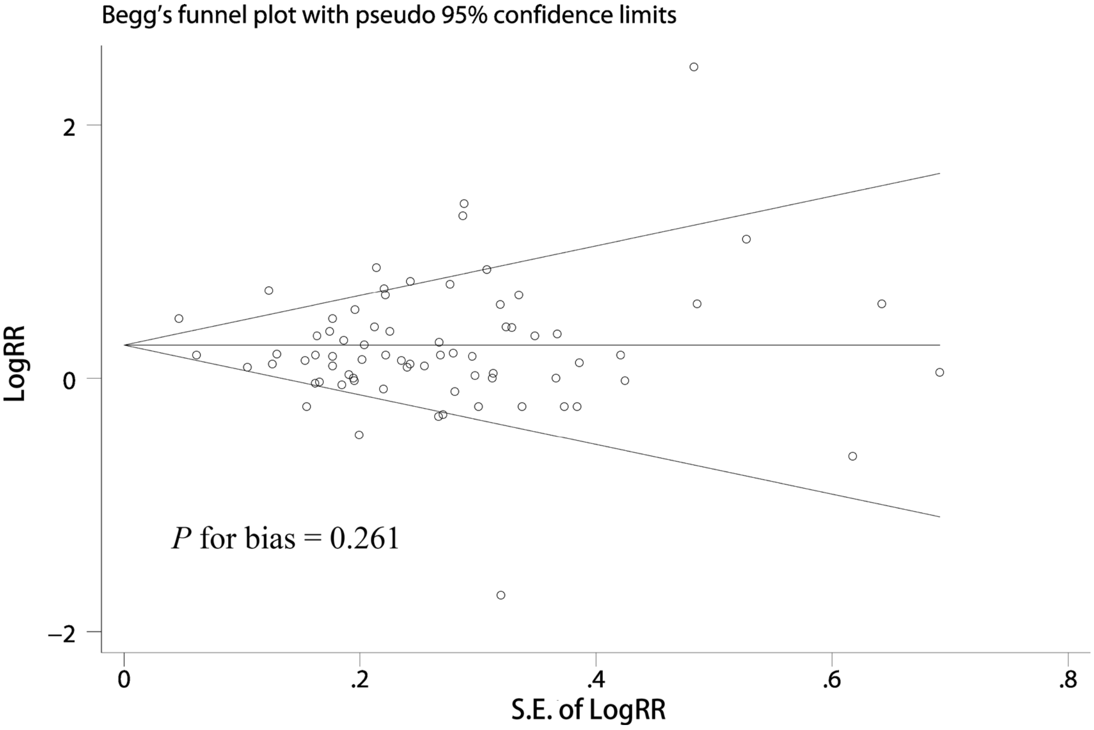

Supplementary Figure 1: Funnel plot of the association between total alcohol drinking and gastric cancer risk. Begg's regression asymmetry test (*P* = 0.261).

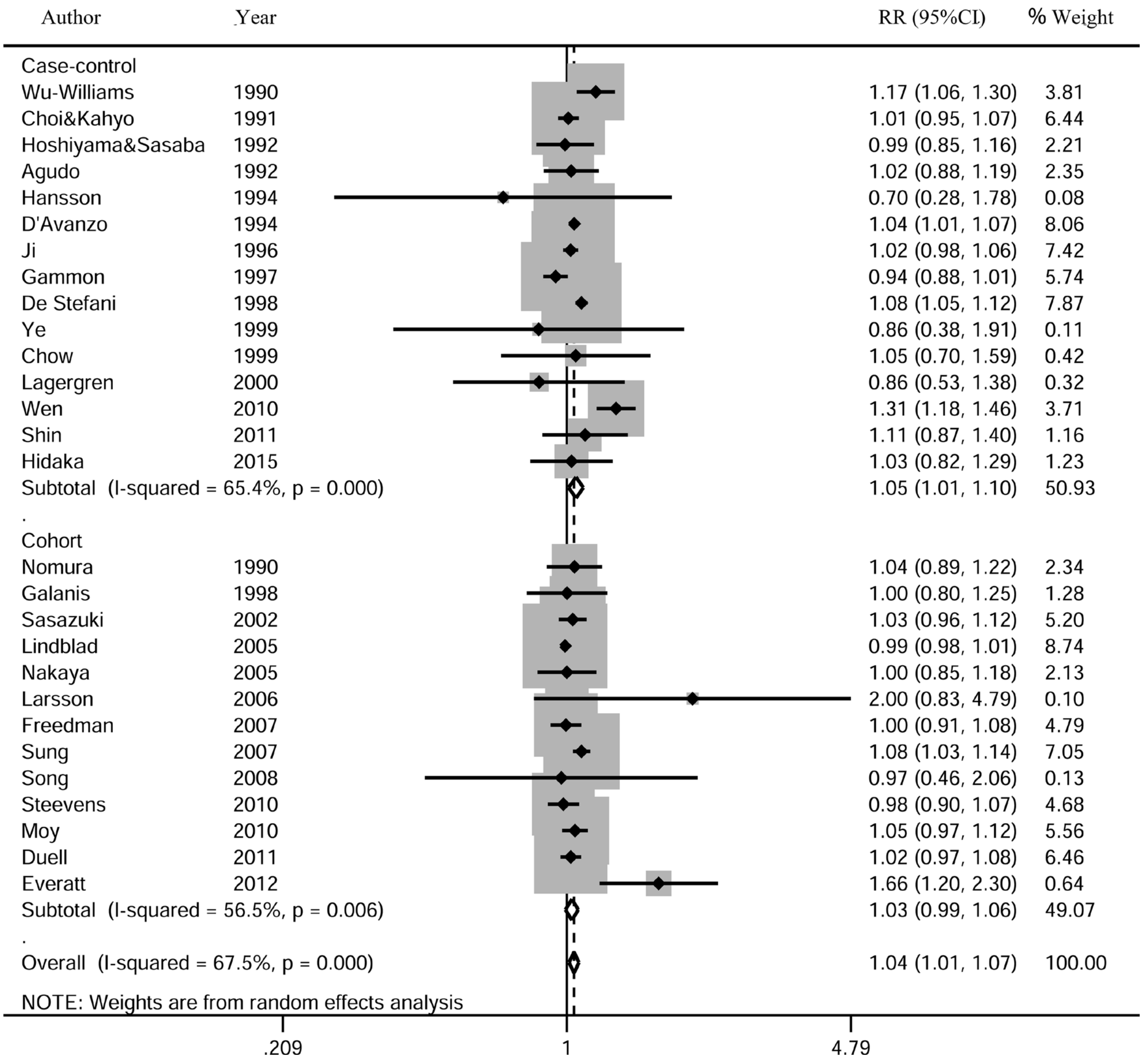

Supplementary Figure 2: Dose-response analysis of per 1 drink/day (12.5 g/day) increase of total alcohol drinking. Studies are grouped according to study design. The pooled RRs were calculated using the random-effects models. Open diamond denote the pooled RR. The size of gray box is positively proportional to the weight assigned to each study (inverse of variance), and horizontal lines represent the 95% confidence intervals. RR, relative risk; CI, confidence interval.

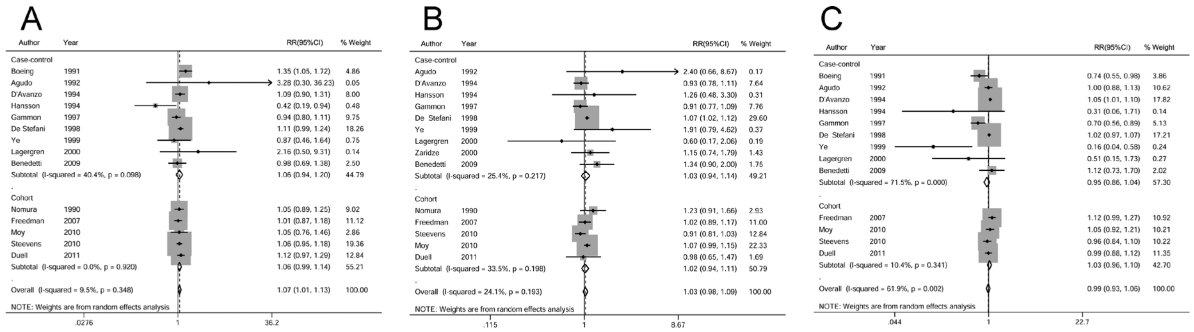

Supplementary Figure 3: Dose-response analyses of per 1 drink/day (12.5g/day) increase of beer (**A**), liquor (**B**), and wine (**C**) drinking. The pooled RRs were calculated using the random-effects models. Open diamond denote the pooled RR. The size of gray box is positively proportional to the weight assigned to each study (inverse of variance), and horizontal lines represent the 95% confidence intervals. RR, relative risk; CI, confidence interval.

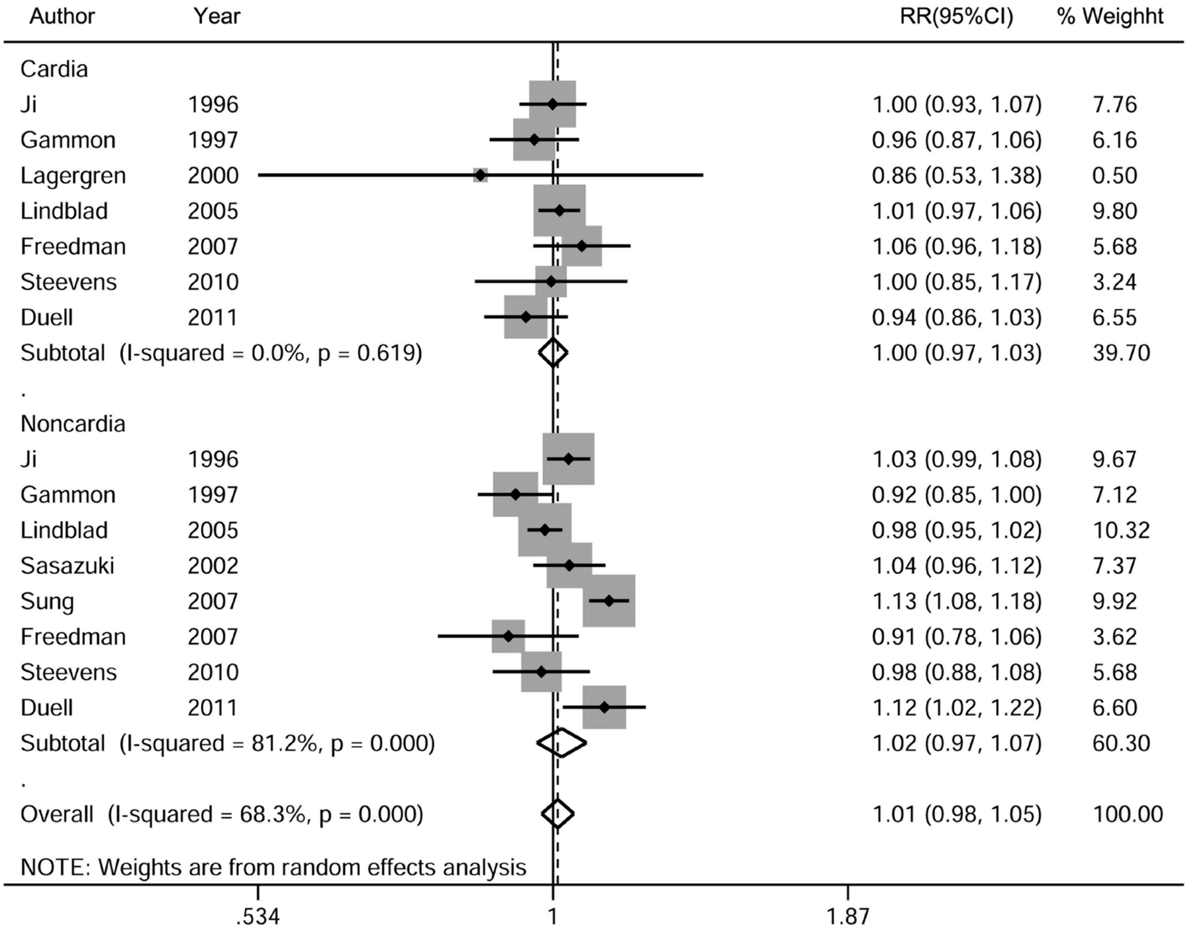

Supplementary Figure 4: Dose-response analysis of per 1 drink/day (12.5g/day) increase of total alcohol drinking stratified by cancer sites. The pooled RRs were calculated using the random-effects models. Open diamond denote the pooled RR. The size of gray box is positively proportional to the weight assigned to each study (inverse of variance), and horizontal lines represent the 95% confidence intervals. RR, relative risk; CI, confidence interval.

Supplementary Table 1: Characteristics of the included studies. See_Supplementary_Table 1

Supplementary Table 2: Quality assessment of included studies according to the Newcastle-Ottawa scale. See_Supplementary_Table 2

EXCLUDED STUDIES

29 studies were excluded after reviewed the full text. 17 studies [1–17] which did not provide relative risk estimate (OR, RR or HR) or corresponding 95% confidence intervals (CIs) were excluded. 4 studies reported the same study populations were excluded [18–21]. 8 studies did not report the right study endpoints were also excluded [22–29].

REFERENCES

1. Baroudi O, Chaaben AB, Mezlini A, Moussa A, Omrane I, Jilson I, Benammar-Elgaaied A, Chabchoub S. Impact of lifestyle factors and nutrients intake on occurrence of gastrointestinal cancer in Tunisian population. Tumour Biol. 2014; 35:5815–22. https://doi.org/10.1007/s13277-014-1771-x.
2. Zhang J, Zhan Z, Wu J, Zhang C, Yang Y, Tong S, Sun Z, Qin L, Yang X, Dong W. Association among polymorphisms in EGFR gene exons, lifestyle and risk of gastric cancer with gender differences in Chinese Han subjects. PLoS One. 2013; 8:e59254. https://doi.org/10.1371/journal.pone.0059254.
3. Cokkinides VE, Bandi P, Siegel RL, Jemal A. Cancer-related risk factors and preventive measures in US Hispanics/Latinos. CA Cancer J Clin. 2012; 62:353–63. https://doi.org/10.3322/caac.21155.
4. Yassibas E, Arslan P, Yalcin S. Evaluation of dietary and life-style habits of patients with gastric cancer: a case-control study in Turkey. Asian Pac J Cancer Prev. 2012; 13:2291–7.
5. Bastos J, Lunet N, Peleteiro B, Lopes C, Barros H. Dietary patterns and gastric cancer in a Portuguese urban population. Int J Cancer. 2010; 127:433–41. https://doi.org/10.1002/ijc.25013.
6. De Stefani E, Deneo-Pellegrini H, Boffetta P, Ronco AL, Aune D, Acosta G, Mendilaharsu M, Brennan P, Ferro G. Dietary patterns and risk of cancer: a factor analysis in Uruguay. Int J Cancer. 2009; 124:1391–7. https://doi.org/10.1002/ijc.24035.
7. Lopez-Abente G, Sanz-Anquela JM, Gonzalez CA. Consumption of wine stored in leather wine bottles and incidence of gastric cancer. Arch Environ Health. 2001; 56:559–61. https://doi.org/10.1080/00039890109602906.
8. Munoz SE, Ferraroni M, La Vecchia C, Decarli A. Gastric cancer risk factors in subjects with family history. Cancer Epidemiol Biomarkers Prev. 1997; 6:137–40.
9. Gray JR, Coldman AJ, MacDonald WC. Cigarette and alcohol use in patients with adenocarcinoma of the gastric cardia or lower esophagus. Cancer. 1992; 69:2227–31.
10. De Stefani E, Correa P, Fierro L, Carzoglio J, Deneo-Pellegrini H, Zavala D. Alcohol drinking and tobacco smoking in gastric cancer. A case-control study. Rev Epidemiol Sante Publique. 1990; 38:297–307.
11. Unakami M, Hara M, Fukuchi S, Akiyama H. Cancer of the gastric cardia and the habit of smoking. Acta Pathol Jpn. 1989; 39:420–4.
12. Li JY, Ershow AG, Chen ZJ, Wacholder S, Li GY, Guo W, Li B, Blot WJ. A case-control study of cancer of the esophagus and gastric cardia in Linxian. Int J Cancer. 1989; 43:755–61.
13. Trichopoulos D, Ouranos G, Day NE, Tzonou A, Manousos O, Papadimitriou C, Trichopoulos A. Diet and cancer of the stomach: a case-control study in Greece. Int J Cancer. 1985; 36:291–7.
14. Hinds MW, Kolonel LN, Lee J, Hirohata T. Associations between cancer incidence and alcohol/cigarette consumption among five ethnic groups in Hawaii. Br J Cancer. 1980; 41:929–40.
15. Williams RR, Horm JW. Association of cancer sites with tobacco and alcohol consumption and socioeconomic status of patients: interview study from the Third National Cancer Survey. J Natl Cancer Inst. 1977; 58:525–47.
16. Graham S, Lilienfeld AM, Tidings JE. Dietary and purgation factors in the epidemiology of gastric cancer. Cancer. 1967; 20:2224–34.

17. Jing JJ, Sun LP, Xu Q, Yuan Y. Effect of ERCC8 tagSNPs and their association with H. pylori infection, smoking, and alcohol consumption on gastric cancer and atrophic gastritis risk. Tumour Biol. 2015; 36:9525–35. https://doi.org/10.1007/s13277-015-3703-9.
18. Larsson SC, Giovannucci E, Wolk A. Coffee consumption and stomach cancer risk in a cohort of Swedish women. Int J Cancer. 2006; 119:2186–9. https://doi.org/10.1002/ijc.22105.
19. Kwak MS, Choi KS, Park S, Park EC. Perceived risk for gastric cancer among the general Korean population: a population-based survey. Psychooncology. 2009; 18:708–15. https://doi.org/10.1002/pon.1458.
20. Duell EJ, Sala N, Travier N, Munoz X, Boutron-Ruault MC, Clavel-Chapelon F, Barricarte A, Arriola L, Navarro C, Sanchez-Cantalejo E, Quiros JR, Krogh V, Vineis P, et al. Genetic variation in alcohol dehydrogenase (ADH1A, ADH1B, ADH1C, ADH7) and aldehyde dehydrogenase (ALDH2), alcohol consumption and gastric cancer risk in the European Prospective Investigation into Cancer and Nutrition (EPIC) cohort. Carcinogenesis. 2012; 33:361–7. https://doi.org/10.1093/carcin/bgr285.
21. Hidaka A, Sasazuki S, Goto A, Sawada N, Shimazu T, Yamaji T, Iwasaki M, Inoue M, Noda M, Tajiri H, Tsugane S. Plasma insulin, C-peptide and blood glucose and the risk of gastric cancer: the Japan Public Health Center-based prospective study. Int J Cancer. 2015; 136:1402–10. https://doi.org/10.1002/ijc.29098.
22. Ferrari F, Reis MA. Study of risk factors for gastric cancer by populational databases analysis. World J Gastroenterol. 2013; 19:9383–91. https://doi.org/10.3748/wjg.v19.i48.9383.
23. Masaki M, Sugimori H, Nakamura K, Tadera M. Dietary patterns and stomach cancer among middle-aged male workers in Tokyo. Asian Pac J Cancer Prev. 2003; 4:61–6.
24. Morita M, Kuwano H, Baba H, Taketomi A, Kohnoe S, Tomoda H, Araki K, Saeki H, Kitamura K, Sugimachi K. Multifocal occurrence of gastric carcinoma in patients with a family history of gastric carcinoma. Cancer. 1998; 83:1307–11.
25. Brown LM, Silverman DT, Pottern LM, Schoenberg JB, Greenberg RS, Swanson GM, Liff JM, Schwartz AG, Hayes RB, Blot WJ, et al. Adenocarcinoma of the esophagus and esophagogastric junction in white men in the United States: alcohol, tobacco, and socioeconomic factors. Cancer Causes Control. 1994; 5:333–40.
26. Pollack ES, Nomura AM, Heilbrun LK, Stemmermann GN, Green SB. Prospective study of alcohol consumption and cancer. N Engl J Med. 1984; 310:617–21. https://doi.org/10.1056/nejm198403083101003.
27. Oiso T. Incidence of stomach cancer and its relation to dietary habits and nutrition in Japan between 1900 and 1975. Cancer Res. 1975; 35:3254–8.
28. Vaughan TL, Davis S, Kristal A, Thomas DB. Obesity, alcohol, and tobacco as risk factors for cancers of the esophagus and gastric cardia: adenocarcinoma versus squamous cell carcinoma. Cancer Epidemiol Biomarkers Prev. 1995; 4:85–92.
29. Lee YC, Hashibe M. Tobacco, alcohol, and cancer in low and high income countries. Ann Glob Health. 2014; 80:378–83. https://doi.org/10.1016/j.aogh.2014.09.010.
