# Supplementary material for: Alcohol drinking and gastric cancer risk: a meta-analysis of observational studies
Source: Oncotarget. 2017 Sep 15;8(58):99013–23. doi: 10.18632/oncotarget.20918 (PMC5716786; doi:10.18632/oncotarget.20918)
Supplement: Supplementary file 2 [file oncotarget-08-99013-s002.docx]

**Supplementary Table 1: Characteristics of the included studies**

| First Author  (Year) | Country | Design | Study period | Sex | Cancer  site | Case (n) | Control/cohort size  (n) | Type of alcohol | Comparison | RR(95%CI)  (Highest vs Lowest) | Adjusted factors |
| --- | --- | --- | --- | --- | --- | --- | --- | --- | --- | --- | --- |
| Menezes[[1](#_ENREF_1)]  (2015) | Brazil | HCC | 2000-2009 | M+W | Stomach | 9177 | 24840 | Total alcohol | current alcohol consumption vs nonconsumer | 1.6 (1.5-1.8) | Age, race, education, marital status, smoking habits, region of residence, year of diagnosis |
| Hidaka[[2](#_ENREF_2)]  (2015) | Japan | PCC | 1990-2004 | M+W | Stomach | 457 | 457 | Total alcohol | 1+ per day and ≥150g/week vs Never to occasional | 1.09 (0.68–1.74) | Smoking status, BMI, total calorie, family history, H pylori infection, atrophy and history of DM |
| Song[[3](#_ENREF_3)]  (2014) | China | HCC | 2012-2013 | M | GCA | 130 | 160 | Total alcohol | ≥1800g-year vs No | 1.79(0.96,3.35) | smoking, dietary habit |
| Sun[[4](#_ENREF_4)]  (2013) | China | PCC | 2005-2007 | M+W | GCA | 470 | 470 | Total alcohol | Drinkers vs Nondrinkers | 2.36(1.29,4.31) | smoking, fast eating,  hot food/moldy food /leftover food taking, family history of tumor, income, education, fresh vegetables and fruits ,BMI |
| Matsuo[[5](#_ENREF_5)]  (2013) | Japan | HCC | 2001-2005 | M+W | Stomach | 697 | 1372 | Total alcohol | Heavy vs Non-drinker | 1.72(1.17,2.52) | PY of smoking, fruit/vegetable intake, family history of gastric cancer, gastric atrophy, *H pylori* status, ALDH2 genotypes |
| Mao[[6](#_ENREF_6)]  (2011) | China | PCC | 2010-2011 | M+W | Stomach | 200 | 200 | Total alcohol | Current vs Never | 1.93(1.25,2.98) |  |
| Gao[[7](#_ENREF_7)]  (2011) | China | PCC | NR | M | GCA+GNCA | 915 | 1514 | Total alcohol  Beer  Wine  Liquor | GCA: Ever vs Never  GNCA: Ever vs Never  GCA: Ever vs Never  GNCA: Ever vs Never  GCA: Ever vs Never  GNCA: Ever vs Never  GCA: <1984 Daily vs Never  GCA: >1984 Daily vs Never  GNCA: <1984 Daily vs Never  GNCA: >1984 Daily vs Never | 1.01(0.80,1.27)  1.29(0.94,1.76)  1.05(0.84,1.32)  1.53(1.14,2.05)  1.06(0.82,1.37)  1.22(0.87,1.69)  0.95(0.63,1.43)  0.94(0.69,1.28)  1.23(0.72,2.12)  1.23(0.81,1.88) | Age  Geographic region |
| Shin[[8](#_ENREF_8)]  (2011) | Korea | HCC | 2003-2008 | M, W  M+W | Stomach | 445 | 370 | Total alcohol | Heavy drinker vs Never/rare drinker | 1.12(0.7,1.81) | Sex , age, HP infection, smoking , education |
| Wen[[9](#_ENREF_9)]  (2010) | China | HCC | 2008-2010 | M+W | Stomach | 300 | 600 | Total alcohol | ≥45.6g/day vs None-drinkers | 3.96(2.25,6.96) |  |
| Jorge[[10](#_ENREF_10)]  (2010) | Brazil | PCC | NR | M+W | Stomach | 150 | 164 | Total alcohol | Drinkers vs Nondrinkers | 3.60(2.05,6.32) |  |
| Nguyen[[11](#_ENREF_11)]  (2010) | Vietnam | HCC | 2004 | M+W | Stomach | 59 | 109 | Total alcohol | ≥2 units/week vs <2 units/week | 1.8(0.5,6.2) | Age, Sex, smoking, , GSTA1, GSTP1, GSTT1, and GSTM1 genotype. |
| Benedetti[[12](#_ENREF_12)]  (2009) | Canada | PCC | Mid-1980s | M+W | Stomach | 215 | 507 | Total alcohol  Beer  Wine  Spirits | 7+weekly vs Never weekly  7+weekly vs Never weekly  7+weekly vs Never weekly  7+weekly vs Never weekly | 1.15(0.72,1.81)  1.08(0.71,1.66)  0.84(0.49,1.42)  1.45(0.90,2.34) | Age, smoking, respondent status, ethnicity, census tract income, years of schooling, time since quitting |
| Lucenteforte[[13](#_ENREF_13)]  (2008) | Italy | HCC | 1997-2007 | M+W | Stomach | 230 | 547 | Total alcohol | Quintiles 5 vs Quintiles 1 | 1.02(0.57,1.83) | Sex, age, education, year of interview, BMI, smoking, family history of stomach cancer, energy intake |
| Suwanrungruang[[14](#_ENREF_14)]  (2008) | Thailand | HCC | 2002-2006 | M | Stomach | 57 | 114 | Total alcohol | Drinker vs Non-drnker | 1.4(0.68,2.66) | Age, region |
| Boccia[[15](#_ENREF_15)]  (2007) | Italy | HCC | NR | M+W | Stomach | 107 | 254 | Total alcohol | Drinker vs Non-drnker | 2.10(1.22,3.60) | age, sex , familiarity for cancer |
| Fei&Xiao[[16](#_ENREF_16)]  (2006) | China | HCC | NR | M+W | GNCA^a^ | 189 | 567 | Total alcohol | Drinker vs Non-drnker | 2.15(1.34,3.47) |  |
| Lacasana-Navarro[[17](#_ENREF_17)]  (2006) | Mexico | HCC | 1994-1996 | M+W | Stomach | 201 | 427 | Total alcohol | Drinker vs Non-drnker | 0.98(0.67,1.44) | Age, sex, energy intake, scholarship, *H pylori* CagA + status, capsaicin intake, MTHFR and dietary intake of fruit, vegetable and animal sources of folate. |
| Shen[[18](#_ENREF_18)]  (2004) | China | PCC | 1997-1998 | M+W | Stomach^[a]^ | 165 | 295 | Total alcohol | Current vs Never | 0.18(0.10,0.35) | Age , gender |
| Nomura[[19](#_ENREF_19)]  (2003) | USA | PCC | 1993-1999 | M+W | ACDS | 300^b^ | 446 | Total alcohol | Drinker vs Non-drnker | 1.2(0.9,1.7) | sex, age, ethnicity |
| Hamada[[20](#_ENREF_20)]  (2002) | Brazil | HCC | 1991-1994 | M+W | Stomach | 96 | 192^[b]^ | Total alcohol | Daily vs <once/month | 1.8(0.7,4.7) | Sex , age, country of birth |
| Nishimoto[[21](#_ENREF_21)]  (2002) | Brazil | HCC | 1991-1994 | M+W | stomach | 236 | 236 | Total alcohol | Daily vs <once/month | 1.1(0.7,1.9) | Sex , age, fruit vegetable intake, race, education, |
| Kikuchi[[22](#_ENREF_22)]  (2002) | Japan | HCC | 1993-1995 | M,W | stomach | 718 | 883 | Total alcohol | Male:1350 alcohol-years vs never-drinker  Female:135 alcohol-years vs never-drinker | 2.45(1.57,3.83)  1.38(0.77,2.48) | Age, smoking, HP status |
| Rao[[23](#_ENREF_23)]  (2002) | India | HCC | 1988-1992 | M+W | stomach | 170 | 2184 | Total alcohol | Drinker vs Non-drnker | 0.8(0.4,1.3) | Age, sex, residence, religion, literacy |
| Wu[[24](#_ENREF_24)]  (2001) | USA | PCC | 1992-1997 | M+W | GCA+ACDS | 720 | 1356 | Total alcohol  Beer  Wine  Hard liquor | GCA:36+drinks/week vs Never  ACDS:36+drinks/week vs None  GCA:15+drinks/week vs None  ACDS:15+drinks/week vs None  GCA:15+drinks/week vs None  ACDS:15+drinks/week vs None  GCA:15+drinks/week vs None  ACDS:15+drinks/week vs None | 1.35(0.8,2.3)  1.35(0.8,2.2)  1.07(0.7,1.7)  1.67(1.1,2.6)  0.77(0.5,1.0)  0.44(0.2,1.2)  1.08(0.7,1.8)  0.7(0.4,1.1) | Smoking, age, sex, race birthplace, education |
| Munoz[[25](#_ENREF_25)]  (2001) | Venezuela | HCC+PCC^[c]^ | 1991-1997 | M | stomach | 207 | 340 | Total alcohol | Current vs Never/occassional | 3.1(2.0,4.6) | Age and SES |
| Chen[[26](#_ENREF_26)]  (2000) | China | HCC | 1992-1996 | M+W | GCA^[d]^ | 152 | 497 | Total alcohol | Drinker vs Non-drnker | 1.5(0.9,3.2) | sex, age, years of schooling, time of hospitalization, SES, smoking |
| Zaridze[[27](#_ENREF_27)]  (2000) | Russia | HCC | 1996-1997 | M,W | Stomach | 448 | 610 | Total alcohol  Sweet wine  vodka | Male: Drinker vs Non-drnker  Female: Drinker vs Non-drnker  Male: Drinker vs Non-drnker  Male: High vs Non-drnker  Female: High vs Non-drnker | 1.9(1.1,3.4)  1.2(0.8,1.8)  1.6(1.0,2.6)  1.7(0.9,3.1)  1.3(0.8,2.2) | Age, education, smoking(M)  Age, education, energy (W) |
| Mathew[[28](#_ENREF_28)]  (2000) | India | HCC | 1988-1991 | M+W | Stomach | 194 | 305 | Total alcohol | Drinker vs Non-drnker | 1.5(1.0,3.2) | Age, sex, religion, education income |
| Lagergren[[29](#_ENREF_29)]  (2000) | Sweden | PCC | 1995-1997 | M+W | GCA | 262 | 820 | Total alcohol  Strong beer  Wine  Hard liquor | >70g/week vs never  >25g/week vs never  >25g/week vs never  >30g/week vs never | 0.9(0.5,1.5)  1.4(0.8,2.4)  0.8(0.5,1.2)  0.9(0.5,1.6) | Age, gender, smoking, educational, BMI, reflux symptoms, fruit, vegetables, energy intake, physical activity |
| Ye[[30](#_ENREF_30)]  (1999) | Sweden | PCC | 1989-1995 | M+W | GCA+ACDS | 514 | 1164 | Total alcohol  Light beer  medium-strong beer  strong beer  Wine  Hard liquor | GCA: >160ml/month vs Non-drinkers  ACDS(I):>160ml/month vs Non-drinkers  ACDS(D):>160ml/month vs Non-drinkers  GCA: ≥2400ml/month vs <400ml/month  ACDS(I): ≥2400ml/month vs <400ml/month  ACDS(D): ≥2400ml/month vs <400ml/month  GCA: ≥400ml/month vs <400ml/month  ACDS(I): ≥400ml/month vs <400ml/month  ACDS(D): ≥400ml/month vs <400ml/month  GCA: Drinker vs Non-drinker  ACDS(I): Drinker vs Non-drinker  ACDS(D): Drinker vs Non-drinker  GCA: ≥600ml/month vs Non-drinker  ACDS(I): ≥600ml/month vs Non-drinker  ACDS(D): ≥600ml/month vs Non-drinker  GCA: ≥320ml/month vs Non-drinker  ACDS(I): ≥320ml/month vs Non-drinker  ACDS(D): ≥320ml/month vs Non-drinker | 0.7(0.3,1.5)  1.2(0.7,1.9)  1.0(0.5,1.8)  1.2(0.7,2.3)  1.0(0.7,1.5)  0.9(0.5,1.5)  0.8(0.5,1.5)  0.9(0.6,1.3)  1.0(0.6,1.6)  0.8(0.4,1.5)  1.1(0.7,1.6)  0.9(0.5,1.4)  0.4(0.2,1.1)  0.5(0.3,1.0)  1.1(0.6,2.3)  1.2(0.5,2.8)  1.5(0.9,2.5)  1.4(0.7,2.8) | Age, gender, residence area, BMI, SES, smoking use of smokeless tobacco, |
| Chow[[31](#_ENREF_31)]  (1999) | Poland | PCC | 1994-1997 | M+W  M,W | Stomach | 464 | 480 | Total alcohol  Beer  Wine  Liquor | ≥7 drinks/week vs Non-drinker  Men: Current drinker vs Non-drinker  Women: Current drinker vs Non-drinker  Men: Current drinker vs Non-drinker  Women: Current drinker vs Non-drinker  Men: Current drinker vs Non-drinker  Women: Current drinker vs Non-drinker | 1.2(0.7,2.0)  0.5(0.3,0.9)  1.4(0.5,4.4)  0.5(0.3,0.8)  1.2(0.6,2.6)  0.4(0.3,0.7)  1.4(0.7,2.9) | Age, education, years lived on a farm, smoking,  family history of cancer |
| Lopez-Carrillo[[32](#_ENREF_32)]  (1998) | Mexico | PCC | NR | M+W | Stomach | 220 | 752 | Total alcohol  Beer  Wine  Distilled | 5g/day vs Abstainers  1 drink vs Non-beer consumers  1 drink vs Non-wine consumers  1 drink vs Non-distilled consumers | 1.93(1.00,3.71)  1.04(0.55,1.94)  2.93(1.27,6.75)  1.83(1.07,3.10) | Age, sex, total calorie intake chili pepper, history of peptic ulcer, SES, smoking fruit, vegetables, salt, meat |
| De Stefani[[33](#_ENREF_33)]  (1998) | Uruguay | HCC | 1992-1996 | M | Stomach | 331 | 622 | Total alcohol  Beer  Wine  Hard liquor | 121g+/day vs Non-drinkers  61-120g/day vs Non-drinkers  121g+/day vs Non-drinkers  121g+/day vs Non-drinkers | 2.4(1.6,3.7)  1.9(0.9,3.7)  0.9(0.4,1.8)  2.1(1.1,3.9) | Age, residence, urban/rural, smoking duration vegetable intake, |
| Gammon[[34](#_ENREF_34)]  (1997) | USA | PCC | 1993-1995 | M+W | GCA+GNCA | 629 | 695 | Total alcohol  Beer  Wine  liquor | GCA: >30 drink/week vs Never  GNCA: >30 drink/week vs Never  GCA: >12 drink/week vs Never  GNCA: >12 drink/week vs Never  GCA: >7 drink/week vs Never  GNCA: >7 drink/week vs Never  GCA: >14 drink/week vs Never  GNCA: >14 drink/week vs Never | 0.7(0.4,1.2)  0.6(0.4,1.0)  0.9(0.6,1.5)  0.9(0.5,1.4)  0.8(0.5,1.5)  0.6(0.3,1.1)  0.8(0.4,1.4)  0.9(0.5,1.6) | Age, sex, geographic center race, BMI, income,  cigarette smoking, |
| Gajalakshmi & Shanta[[35](#_ENREF_35)]  (1996) | India | HCC | 1988-1990 | M+W | Stomach | 388 | 388 | Total alcohol  Toddy  Arrack  Foreign liquor^[e]^ | Current drinkers vs Non-drinkers  Ex & current drinker vs Non-drinkers  Ex & current drinker vs Non-drinkers  Ex & current drinker vs Non-drinkers | 0.8(0.41,1.77)  0.4(0.09,2.20)  2.6(1.49,4.40)  3.0(1.49,5.96) | Sex, age, religion, mother tongue, income, educational, area of residence, tobacco chewing, consumption of roots and tubers, fried eggs, chillies and chutney |
| Zhang[[36](#_ENREF_36)]  (1996) | USA | HCC | 1992-1994 | M+W | ACDS | 67 | 132 | Total alcohol  Beer  Wine  Liquor | >1/week vs No  >1/week vs No  >1/week vs No  >1/week vs No | 0.98(0.43,2.27)  1.43(0.45,4.58)  0.97(0.36,2.58)  0.66(0.22,1.99) | Age, sex, race, education smoking, BMI total dietary intake of calories |
| Ji[[37](#_ENREF_37)]  (1996) | China | PCC | 1988-1989 | M | Stomach | 770 | 819 | Total alcohol | ≥525g/week vs Non-drinker | 1.19(0.84,1.68) | age, income, education, and smoking |
| Lee[[38](#_ENREF_38)]  (1995) | Korea | HCC | 1990-1991 | M+W | Stomach | 213 | 211 | Total alcohol | Heavy vs Never or light` | 0.8(0.4,1.5) | Age, sex, education, economic status, residence. |
| Falcao[[39](#_ENREF_39)]  (1994) | Portugal | HCC |  | M+W | Stomach | 74 | 193 | Total alcohol  Beer  Red wine  white wine  Spirts | >560g/week vs Unexposed  >139g/week vs Unexposed  ≤560g/week vs Unexposed  ≤560g/week vs Unexposed  >76g/week vs Unexposed | 1.42(0.69,2.91)  0.56(0.18,0.74)  3.00(1.27,7.09)  0.69(0.13,3.53)  1.67(0.8,3.49) |  |
| Hansson[[40](#_ENREF_40)]  (1994) | Sweden | PCC | 1989-1992 | M+W | Stomach | 338 | 679 | Total alcohol  Light beer  medium-strong beer  strong beer  Wine  Hard liquid | >160ml/month vs Non-drinkers  ≥2400ml/month vs <400ml/month  ≥400ml/month vs <400ml/month  Drinker vs Non-drinker  ≥600ml/month vs Non-drinker  ≥320ml/month vs Non-drinker | 0.92(0.60,1.42)  0.76(0.51,1.13)  0.96(0.68,1.37)  0.95(0.67,1.35)  0.57(0.31,1.04)  1.27(0.83,1.96) | Age, sex , SES |
| Inoue[[41](#_ENREF_41)]  (1994) | Japan | HCC | 1988-1991 | M+W  M,W | Stomach | 668 | 668 | Total alcohol | Drinker vs Non-drinker | 1.12(0.88,1.44) | Sex, age, time of hospital visit |
| D’Avanzo[[42](#_ENREF_42)]  (1994) | Italy | HCC | 1985-1993 | M+W  M,W | Stomach | 746 | 2053 | Total alcohol  Beer  Wine  Spirits | ≥8 drinks/day vs Nondrinkers  ≥2 drinks/day vs Nondrinkers  ≥8 drinks/day vs Nondrinkers  ≥2 drinks/day vs Nondrinkers | 1.6(1.1,2.2)  1.1(0.7,1.9)  1.4(1.0,2.0)  0.9(0.5,1.5) | Sex Age |
| Jedrychowski[[43](#_ENREF_43)]  (1993) | Poland | HCC | 1986-1990 | M | Stomach | 520 | 520 | Vodka | ≥1/week vs Non-drinker | 3.06(1.90,4.95) | age, sex, occupation, education, sausage/fruit/vegetable consumption, smoking |
| Kabat[[44](#_ENREF_44)]  (1993) | USA | HCC | 1981-1990 | M,W | ACDS | 143 | 6384 | Total alcohol | Male: 4+oz w-e/d vs nondrinker  Female: 4+oz w-e/d vs nondrinker | 0.7(0.4,1.3)  0.9(0.3,3.1) | Age, education, smoking hospital, time period |
| Hoshiyama & Sasaba[[45](#_ENREF_45)]^[f]^  (1992) | Japan | HCC,PCC | 1984-1990 | M+W | Stomach | 294 | 294(PCC)  202(HCC) | Total alcohol | ≥ 50ml/day vs Never | 1.0(0.5,1.7) | Sex, age, administrative division, smoking |
| Agudo[[46](#_ENREF_46)]  (1992) | Spain | HCC | 1987-1989 | M,W | Stomach | 354 | 354 | Total alcohol  Beer  wine  Liquors  Aperitifs | Men: >36g/day vs Nonconsumers  Women: 19-36g/day vs Nonconsumers  ＞100cc/day vs Nonconsumers  ＞300cc/day vs Nonconsumers  ＞22cc/day vs Nonconsumers  Consumers vs Nonconsumers | 1.22(0.68,2.19)  1.24(0.25,6.28)  1.78(0.55,5.75)  0.98(0.65,1.80)  2.07(0.69,6.24)  0.58(0.05,6.24) | Age residence, fruits consumption, cooked vegetables, cold cuts preserved fish, caloric intake |
| Choi & Kahyo[[47](#_ENREF_47)]  (1991) | Korea | HCC | 1986-1990 | M | Stomach | 238 | 714 | Total alcohol | Heavy vs Non-drinker | 1.19(0.67,2.13) | age, marital status, education, diet, smoking |
| Tominaga[[48](#_ENREF_48)]  (1991) | Japan | HCC | 1971-1985 | M+W | Stomach | 294 | 588 | Total alcohol | Daily vs Nondrinker | 1.16(0.78,1.72) | Sex, age, area of residence |
| Yu&Hsieh[[49](#_ENREF_49)]  (1991) | China | PCC | 1976-1980 | M+W | Stomach | 84 | 2676 | Total alcohol | Users vs Nonusers | 0.8(0.4,1.8) | Age, sex, family income, family history of stomach cancer, family history of other cancer, history of tuberculosis, blood type, smoking, strong tea, fruit milk consumption |
| Boeing[[50](#_ENREF_50)]  (1991) | Germany | HCC | 1985-1988 | M+W | Stomach | 143 | 579 | Beer  Wine  Liquor | ＞500g/day vs No consumption  ＞20g/day vs No consumption  ＞2g/day vs No consumption | 1.82(0.95,3.50)  0.52(0.30,0.93)  0.52(0.27,1.00) | Age, Sex, Hospital |
| Wu-Williams[[51](#_ENREF_51)]  (1990) | USA | PCC | 1975-1982 | M | Stomach | 137 | 137 | Total alcohol  Beer  Sweet wine  Dinner wine  Hard liquor | ≥ 80g/day vs Nondrinker  Daily vs Nondrinker  Daily vs Nondrinker  Daily vs Nondrinker  Daily vs Nondrinker | 3.0(1.1,8.7)  3.5(1.4,8.7)  2.9(0.5,18.0)  1.6(0.6,4.3)  2.3(0.9,5.6) | Age, race |
| Buiatti[[52](#_ENREF_52)]  (1990) | Italy | PCC | 1985-1987 | M+W | Stomach | 1016 | 1159 | Total alcohol | Quintiles 5 vs Quintiles 1 | 1.4(1.0,1.9) | age, sex, area, place of residence, migration from south, SES, familial GC history, Quetelet index, energy intake |
| Kato[[53](#_ENREF_53)]  (1990) | Japan | HCC | 1985-1989 | M,W | Stomach | 427 | 3014 | Total alcohol | Males: Daily vs Non-drinkers  Females: Daily vs Non-drinkers | 0.99(0.71,1.37)  0.73(0.26,2.08) | Age, residence. |
| Hu[[54](#_ENREF_54)]  (1988) | China | HCC | 1985-1986 | M+W | Stomach | 241 | 241 | Total alcohol | ≥4kg/year vs <4kg/year | 1.45(1.02,2.02) | Sex, age, smoking  Chinese cabbage intake |
| You[[55](#_ENREF_55)]  (1988) | China | PCC | 1984-1986 | M | Stomach | 443 | 888 | Total alcohol | ≥365 times/year vs 0 | 0..8(0.6,1.1) | Age, family income. Smoking |
| Correa[[56](#_ENREF_56)]  (1985) | USA | HCC | 1979-1983 | M+W | Stomach | 391 | 391 | Total alcohol  Beer  Wine  Hard liquor | Blacks: high vs low  Whites: high vs low  Blacks :Drinkers vs Non-drinkers  Whites :Drinkers vs Non-drinkers  Blacks :Drinkers vs Non-drinkers  Whites :Drinkers vs Non-drinkers  Blacks :Drinkers vs Non-drinkers  Whites :Drinkers vs Non-drinkers | 2.21(1.19,4.08)  1.87(1.02,3.44)  1.27(0.82,1.99)  1.17(0.72,1.90)  1.18(0.51,2.73)  1.61(0.89.2.91)  1.54(0.94,2.54)  1.99(1.21,3.28) | Sex, age, race, respondent status, Income |
| Tuyns[[57](#_ENREF_57)]  (1982) | France | HCC | 1973-1980 | M+W | Stomach | 163 | 1976 | Total alcohol | Consumers vs Non-consumers | 0.54(0.16,1.80) | Sex age |
| Hoey[[58](#_ENREF_58)]  (1981) | France | HCC | 1978-1980 | M | Stomach | 40 | 168 | Total alcohol | Drinkers vs Non-drinkers | 4.7(11.7,31.2) | Age, lettuce |
| Ma[[59](#_ENREF_59)]  (2015) | Korea | Cohort | 1993-2004 | M+W | Stomach | 403 | 18863PR | Total alcohol | Current vs Non-drinking | 1.21(0.94,1.56) | Age, sex, BMI, education level, smoking status |
| Jayalekshmi[[60](#_ENREF_60)]  (2015) | India | Cohort | 1990-2009 | M | Stomach | 116 | 65553PR  900721PY | Total alcohol | Current vs Never | 1.3(0.9,2.0) |  |
| Everatt[[61](#_ENREF_61)]  (2012) | Lithuania | Cohort | 1978-2008 | M | stomach | 185 | 7150PR^[g]^  137187PY | Total alcohol  beer  wine  vodka | ≥100g/week vs Non-drinkers  ≥1 litre vs Non-drinkers  ≥0.5 litre vs Non-drinkers  ≥200 g vs Non-drinkers | 2.00(1.04,3.82) vs 0.95(0.47,1.93)  0.79(0.39,1.62) vs 0.83(0.58,1.18)  2.95(1.30,6.68) vs 1.47(0.87,2.50)  1.25(0.82,1.92) vs 1.20(0.63,2.25) | Smoking, education, BMI |
| Duell[[62](#_ENREF_62)]  (2011) | Europe^[h]^ | Cohort | 1992/1998-2002/2004 | M+W | stomach | 444 | 478459PR  4160578PY | Total alcohol  beer  wine  liquor and spirits | ≥60g/day vs Never  ≥30g/day vs Never  ≥30g/day vs Never  ≥10g/day vs Never | 1.65(1.06,2.58) vs 1.37(1.01,1.86)  1.75(1.13,2.73) vs 1.14(0.88,1.48)  0.89(0.60,1.30) vs 1.00(0.76,1.32)  1.08(0.71,1.63) vs 1.02(0.79,1.32) | age, sex, center, education, smoking, intake of fruit/nuts/seeds, vegetables, processed and red meat, total energy |
| Moy[[63](#_ENREF_63)]  (2010) | China | Cohort | 1986/1989-2005 | M | stomach | 391 | 18244PR  281808PY | Total alcohol  Beer  rice wine  spirits | 40+g/day vs Nondrinkers  1+drink/day vs Nondrinkers  2+drink/day vs Nondrinkers  4+drink/day vs Nondrinkers | 1.15(0.85,1.55)  1.21(0.79,1.84)  1.14(0.80,1.63)  1.40(0.92,2.14) | education, BMI smoking, intake of preserved food items, fresh fruits/ vegetables |
| Steevens[[64](#_ENREF_64)]  (2010) | Netherland | Cohort | 1986-2002  Average Cohort 16.3 years of follow-up | M+W | GCA+GNCA | 164(GCA)  491(GNCA) | 3962PR  56806PY | Total alcohol  beer  wine  liquor | GCA:≥30g/day vs Abstainer  GNCA:≥30g/day vs Abstainer  GCA:＞2glasses/day vs no beer  GNCA:＞2glasses/day vs no beer  GCA:＞2glasses/day vs no wine  GNCA:＞2glasses/day vs no wine  GCA:＞2glasses/day vs no liquor  GNCA:＞2glasses/day vs no liquor | 0.90(0.50,1.64)  1.00(0.68,1.47)  0.90(0.39,2.07)  1.58(0.95,2.63)  1.04(0.40,2.70)  0.88(0.48,1.63)  0.72(0.24,2.18)  0.58(0.33,1.03) | age, sex, smoking, , BMI , education, intake of energy, fruits, vegetables, fish |
| Song[[65](#_ENREF_65)]  (2008) | Korea | Cohort | 1993/1998-2004 | M,W | stomach | 151 | 13396PR  116997.1PY | Total alcohol | Male:＞110g/week vs ≤12g/week  Female:＞110g/week vs ≤12g/week | 0.56(0.29,1.09)  2.23(0.79,6.29) | smoking, salty food, spicy food |
| Sung[[66](#_ENREF_66)]  (2007) | Korea | Cohort | 1996-2002  Average 6.5 years of follow-up | M | stomach | 3452 | 669570PR  4353317PY | Total alcohol | ≥25g/day vs 0 g/day | 1.2(1.1,1.4) | age, BMI, smoking, preference for saltiness in food |
| Freedman[[67](#_ENREF_67)]  (2007) | USA | Cohort | 1995/1996-2000  Average follow up 4.6Y | M+W | GCA+GNCA | 188(GCA)  187(GNCA) | 474606PR  2121797PY | Total alcohol  beer  wine  liquor | GCA:＞3drinks/day vs 0 drink/day  GNCA:＞3drinks/day vs 0 drink/day  GCA:＞3drinks/day vs 0 drink/day  GNCA:＞3drinks/day vs 0 drink/day  GCA:＞3drinks/day vs 0 drink/day  GNCA:＞3drinks/day vs 0 drink/day  GCA:＞3drinks/day vs 0 drink/day  GNCA:＞3drinks/day vs 0 drink/day | 1.57(0.98,2.52) vs 1.19(0.83,1.70)  0.62(0.30,1.27) vs 1.30(0.93,1.82)  1.17(0.55,2.52) vs 1.35(0.94,1.94)  1.02(0.40,2.61) vs 1.18(0.83,1.70)  3.01(0.73,12.31) vs 0.73(0.51,1.05)  4.29(1.05,17.61) vs 1.04(0.73,1.48)  2.15(1.20,3.87) vs 1.36(0.94,2.00)  0.27(0.07,1.10) vs 1.03(0.72,1.47) | age , sex , intake of fruit, vegetable, energy, BMI , education, smoke, physical activity |
| Larsson[[68](#_ENREF_68)]^[i]^  (2006) | Sweden | Cohort | 1987/1990-2005 | W | stomach | 160 | 61433PR  966807PY | Total alcohol  light beer  medium-strong/  strong beer  wine  hard liquor | ≥40.0g/week vs Nondrinkers  ＞2 servings/week vs Nondrinkers  ＞1 servings/week vs Nondrinkers  ＞0.5 servings/week vs Nondrinkers  ＞0.5 servings/week vs Nondrinkers | 1.33(0.79,2.25)  0.91(0.57,1.46)  2.09(1.11,3.93)  0.98(0.58,1.66)  1.38(0.72,2.65) | age , education, intake of fruit and vegetable, processed meat, coffee |
| Sjodahl[[69](#_ENREF_69)]  (2006) | Norway | Cohort | 1984/1986-2002  Average follow-up 16 years | M+W | stomach | 251 | 69962PR  1117648PY | Total alcohol | ≥5 times vs Never drinking | 1.49(0.78,2.83) | Sex, education, BMI, smoking |
| Barstad[[70](#_ENREF_70)]  (2005) | Denmark | Cohort | 1964-1997 | M+W | stomach | 122 | 28463PR  389051PY | Total alcohol  beer  wine  spirits | 28+drinks/week vs < 1 drink/week  13+drinks/week vs < 1 drink/week  13+drinks/week vs < 1 drink/week  13+drinks/week vs < 1 drink/week | 1.13(0.41,1.86)  NR  0.16(0.02,1.18)  1.99(0.88,4.52) | age, sex, smoking |
| Nakaya[[71](#_ENREF_71)]  (2005) | Japan | Cohort | 1990-1997 | M | stomach | 247 | 21201PR 153389PY | Total alcohol | ≥22.8g/day vs Never drinkers | 1.0(0.7,1.5) | age ; smoking; education; consumption of orange, other fruit juice, spinach, carrot or pumpkin, tomato |
| Lindblad[[72](#_ENREF_72)]  (2005) | UK | Nested | 1994-2001 | M+W | Stomach | 1023 | 10000 | Total alcohol | >34 units/day vs 0-2 units/day | 0.75(0.44,1.27) | sex, age, calendar year, smoking, BMI, reflux history |
| Sasazuki[[73](#_ENREF_73)]  (2002) | Japan | Cohort | 1990-1999 | M | stomach | 293 | 19657PR | Total alcohol | 322.5+g/week vs 0-3 days/month | 1.1(0.8,1.6) | age, area, smoking  intake of fruit, green or yellow vegetables, salted cod roe or fish gut, BMI |
| Galanis[[74](#_ENREF_74)]  (1998) | USA | Cohort | 1975/1980-1994  Average follow-up 14.8 years | M | stomach | 64 | 5546PR | Total alcohol | 3+drinks/day vs Non-drinker | 1.2(0.5,2.6) | age, education, place of birth, smoking |
| Nomura[[75](#_ENREF_75)]  (1990) | USA | Cohort | 1965/1968-1986 | M | stomach | 150 | 7990PR  140190PY | Total alcohol  beer  wine  spirits | ≥40 oz/month vs Nondrinker  ≥500 oz/month vs Nondrinker  ≥2 oz/month vs Nondrinker  ≥50 oz/month vs Nondrinker | 1.1(0.7,1.9)  1.1(0.7,1.7)  0.7(0.4,1.3)  1.0(0.5,2.1) | Age, smoking |

HCC: hospital-based case-control study, PCC: population-based case-control study, Nested: nested case-control study, GCA: gastric cardia adenocarcinoma, GNCA: gastric noncardia adenocarcinoma, ACDS: adenocarcinoma of distal stomach, I: intestinal type, D: diffuse type, M: man, W: woman, M+W: man and woman performed together, BMI: body mass index, SES: socioeconomic status, PR: persons at risk, PY: person-year, NR: not reported

a: 57 gastric cardia cancer cases were included.

b: The control group excluded 80 health volunteers.

c: The control group included both hospital-based and population-based participants.

d:Cases with gastric non-cardia cancer were excluded.

e:Foreign liquor included wine, whisky, beer, brandy, gin, and rum.

f:The data of population-based control group were used.

g: The study contains 2 cohorts, that is, one cohort with2447 men from Kaunas Rotterdam Intervention Study (KRIS), and the other with 5933 men from Multifactorial Ischemic Heart Disease Prevention Study (MIHDPS).

h: Thestudy was a multicenter cohort study, in which the participants were from 10 European countries (i.e. Denmark, France, Germany, Greece, Italy, Netherlands, Norway, Spain, Sweden, and the United Kingdom).

I : The analysis was based on the data of the longer period of follow-up (1987-2005).

**References:**

1. de Menezes RF, Bergmann A, de Aguiar SS, Thuler LC. Alcohol consumption and the risk of cancer in Brazil: A study involving 203,506 cancer patients. Alcohol. 2015; 49: 747-51. doi: 10.1016/j.alcohol.2015.07.001.

2. Hidaka A, Sasazuki S, Matsuo K, Ito H, Sawada N, Shimazu T, Yamaji T, Iwasaki M, Inoue M, Tsugane S. Genetic polymorphisms of ADH1B, ADH1C and ALDH2, alcohol consumption, and the risk of gastric cancer: the Japan Public Health Center-based prospective study. Carcinogenesis. 2015; 36: 223-31. doi: 10.1093/carcin/bgu244.

3. Song Q, Hu P, Wang J, Jia Y, Zhang G, Lv L, Liu Y, Cheng Y. Association between gastric cardia adenocarcinoma risk and alcohol flushing response, but not alcohol consumption. Med Oncol. 2014; 31: 858. doi: 10.1007/s12032-014-0858-y.

4. Sun CQ, Chang YB, Cui LL, Chen JJ, Sun N, Zhang WJ, Jia XC, Tian Y, Dai LP. A population-based case-control study on risk factors for gastric cardia cancer in rural areas of Linzhou. Asian Pac J Cancer Prev. 2013; 14: 2897-901.

5. Matsuo K, Oze I, Hosono S, Ito H, Watanabe M, Ishioka K, Ito S, Tajika M, Yatabe Y, Niwa Y, Yamao K, Nakamura S, Tajima K, et al. The aldehyde dehydrogenase 2 (ALDH2) Glu504Lys polymorphism interacts with alcohol drinking in the risk of stomach cancer. Carcinogenesis. 2013; 34: 1510-5. doi: 10.1093/carcin/bgt080.

6. Mao XQ, Jia XF, Zhou G, Li L, Niu H, Li FL, Liu HY, Zheng R, Xu N. Green tea drinking habits and gastric cancer in southwest China. Asian Pac J Cancer Prev. 2011; 12: 2179-82.

7. Gao Y, Hu N, Han XY, Ding T, Giffen C, Goldstein AM, Taylor PR. Risk factors for esophageal and gastric cancers in Shanxi Province, China: a case-control study. Cancer Epidemiol. 2011; 35: e91-9. doi: 10.1016/j.canep.2011.06.006.

8. Shin CM, Kim N, Cho SI, Kim JS, Jung HC, Song IS. Association between alcohol intake and risk for gastric cancer with regard to ALDH2 genotype in the Korean population. Int J Epidemiol. 2011; 40: 1047-55. doi: 10.1093/ije/dyr067.

9. Wen XY. Salt taste sensitivity, physical activity and gastric cancer. Asian Pac J Cancer Prev. 2010; 11: 1473-7.

10. Jorge YC, Duarte MC, Silva AE. Gastric cancer is associated with NOS2 -954G/C polymorphism and environmental factors in a Brazilian population. BMC Gastroenterol. 2010; 10: 64. doi: 10.1186/1471-230x-10-64.

11. Nguyen TV, Janssen MJ, van Oijen MG, Bergevoet SM, te Morsche RH, van Asten H, Laheij RJ, Peters WH, Jansent JB. Genetic polymorphisms in GSTA1, GSTP1, GSTT1, and GSTM1 and gastric cancer risk in a Vietnamese population. Oncol Res. 2010; 18: 349-55.

12. Benedetti A, Parent ME, Siemiatycki J. Lifetime consumption of alcoholic beverages and risk of 13 types of cancer in men: results from a case-control study in Montreal. Cancer Detect Prev. 2009; 32: 352-62.

13. Lucenteforte E, Scita V, Bosetti C, Bertuccio P, Negri E, La Vecchia C. Food groups and alcoholic beverages and the risk of stomach cancer: a case-control study in Italy. Nutr Cancer. 2008; 60: 577-84. 10.1080/01635580802054512.

14. Suwanrungruang K, Sriamporn S, Wiangnon S, Rangsrikajee D, Sookprasert A, Thipsuntornsak N, Satitvipawee P, Poomphakwaen K, Tokudome S. Lifestyle-related risk factors for stomach cancer in northeast Thailand. Asian Pac J Cancer Prev. 2008; 9: 71-5.

15. Boccia S, Sayed-Tabatabaei FA, Persiani R, Gianfagna F, Rausei S, Arzani D, La Greca A, D'Ugo D, La Torre G, van Duijn CM, Ricciardi G. Polymorphisms in metabolic genes, their combination and interaction with tobacco smoke and alcohol consumption and risk of gastric cancer: a case-control study in an Italian population. BMC Cancer. 2007; 7: 206. doi: 10.1186/1471-2407-7-206.

16. Fei SJ, Xiao SD. Diet and gastric cancer: a case-control study in Shanghai urban districts. Chin J Dig Dis. 2006; 7: 83-8. doi: 10.1111/j.1443-9573.2006.00252.x.

17. Lacasana-Navarro M, Galvan-Portillo M, Chen J, Lopez-Cervantes M, Lopez-Carrillo L. Methylenetetrahydrofolate reductase 677C>T polymorphism and gastric cancer susceptibility in Mexico. Eur J Cancer. 2006; 42: 528-33. doi: 10.1016/j.ejca.2005.10.020.

18. Shen J, Wang RT, Wang LW, Xu YC, Wang XR. A novel genetic polymorphism of inducible nitric oxide synthase is associated with an increased risk of gastric cancer. World J Gastroenterol. 2004; 10: 3278-83.

19. Nomura AM, Hankin JH, Kolonel LN, Wilkens LR, Goodman MT, Stemmermann GN. Case-control study of diet and other risk factors for gastric cancer in Hawaii (United States). Cancer Causes Control. 2003; 14: 547-58.

20. Hamada GS, Kowalski LP, Nishimoto IN, Rodrigues JJ, Iriya K, Sasazuki S, Hanaoka T, Tsugane S. Risk factors for stomach cancer in Brazil (II): a case-control study among Japanese Brazilians in Sao Paulo. Jpn J Clin Oncol. 2002; 32: 284-90.

21. Nishimoto IN, Hamada GS, Kowalski LP, Rodrigues JG, Iriya K, Sasazuki S, Hanaoka T, Tsugane S. Risk factors for stomach cancer in Brazil (I): a case-control study among non-Japanese Brazilians in Sao Paulo. Jpn J Clin Oncol. 2002; 32: 277-83.

22. Kikuchi S, Nakajima T, Kobayashi O, Yamazaki T, Kikuichi M, Mori K, Oura S, Watanabe H, Nagawa H, Otani R, Okamoto N, Kurosawa M, Anzai H, et al. U-shaped effect of drinking and linear effect of smoking on risk for stomach cancer in Japan. Jpn J Cancer Res. 2002; 93: 953-9.

23. Rao DN, Ganesh B, Dinshaw KA, Mohandas KM. A case-control study of stomach cancer in Mumbai, India. Int J Cancer. 2002; 99: 727-31. doi: 10.1002/ijc.10339.

24. Wu AH, Wan P, Bernstein L. A multiethnic population-based study of smoking, alcohol and body size and risk of adenocarcinomas of the stomach and esophagus (United States). Cancer Causes Control. 2001; 12: 721-32.

25. Munoz N, Plummer M, Vivas J, Moreno V, De Sanjose S, Lopez G, Oliver W. A case-control study of gastric cancer in Venezuela. Int J Cancer. 2001; 93: 417-23.

26. Chen MJ, Chiou YY, Wu DC, Wu SL. Lifestyle habits and gastric cancer in a hospital-based case-control study in Taiwan. Am J Gastroenterol. 2000; 95: 3242-9. doi: 10.1111/j.1572-0241.2000.03260.x.

27. Zaridze D, Borisova E, Maximovitch D, Chkhikvadze V. Alcohol consumption, smoking and risk of gastric cancer: case-control study from Moscow, Russia. Cancer Causes Control. 2000; 11: 363-71.

28. Mathew A, Gangadharan P, Varghese C, Nair MK. Diet and stomach cancer: a case-control study in South India. Eur J Cancer Prev. 2000; 9: 89-97.

29. Lagergren J, Bergstrom R, Lindgren A, Nyren O. The role of tobacco, snuff and alcohol use in the aetiology of cancer of the oesophagus and gastric cardia. Int J Cancer. 2000; 85: 340-6.

30. Ye W, Ekstrom AM, Hansson LE, Bergstrom R, Nyren O. Tobacco, alcohol and the risk of gastric cancer by sub-site and histologic type. Int J Cancer. 1999; 83: 223-9.

31. Chow WH, Swanson CA, Lissowska J, Groves FD, Sobin LH, Nasierowska-Guttmejer A, Radziszewski J, Regula J, Hsing AW, Jagannatha S, Zatonski W, Blot WJ. Risk of stomach cancer in relation to consumption of cigarettes, alcohol, tea and coffee in Warsaw, Poland. Int J Cancer. 1999; 81: 871-6.

32. Lopez-Carrillo L, Lopez-Cervantes M, Ramirez-Espitia A, Rueda C, Fernandez-Ortega C, Orozco-Rivadeneyra S. Alcohol consumption and gastric cancer in Mexico. Cad Saude Publica. 1998; 14 Suppl 3: 25-32.

33. De Stefani E, Boffetta P, Carzoglio J, Mendilaharsu S, Deneo-Pellegrini H. Tobacco smoking and alcohol drinking as risk factors for stomach cancer: a case-control study in Uruguay. Cancer Causes Control. 1998; 9: 321-9.

34. Gammon MD, Schoenberg JB, Ahsan H, Risch HA, Vaughan TL, Chow WH, Rotterdam H, West AB, Dubrow R, Stanford JL, Mayne ST, Farrow DC, Niwa S, et al. Tobacco, alcohol, and socioeconomic status and adenocarcinomas of the esophagus and gastric cardia. J Natl Cancer Inst. 1997; 89: 1277-84.

35. Gajalakshmi CK, Shanta V. Lifestyle and risk of stomach cancer: a hospital-based case-control study. Int J Epidemiol. 1996; 25: 1146-53.

36. Zhang ZF, Kurtz RC, Sun M, Karpeh M, Jr., Yu GP, Gargon N, Fein JS, Georgopoulos SK, Harlap S. Adenocarcinomas of the esophagus and gastric cardia: medical conditions, tobacco, alcohol, and socioeconomic factors. Cancer Epidemiol Biomarkers Prev. 1996; 5: 761-8.

37. Ji BT, Chow WH, Yang G, McLaughlin JK, Gao RN, Zheng W, Shu XO, Jin F, Fraumeni JF, Jr., Gao YT. The influence of cigarette smoking, alcohol, and green tea consumption on the risk of carcinoma of the cardia and distal stomach in Shanghai, China. Cancer. 1996; 77: 2449-57. doi: 10.1002/(sici)1097-0142(19960615)77:12<2449::aid-cncr6>3.0.co;2-h.

38. Lee JK, Park BJ, Yoo KY, Ahn YO. Dietary factors and stomach cancer: a case-control study in Korea. Int J Epidemiol. 1995; 24: 33-41.

39. Falcao JM, Dias JA, Miranda AC, Leitao CN, Lacerda MM, da Motta LC. Red wine consumption and gastric cancer in Portugal: a case-control study. Eur J Cancer Prev. 1994; 3: 269-76.

40. Hansson LE, Baron J, Nyren O, Bergstrom R, Wolk A, Adami HO. Tobacco, alcohol and the risk of gastric cancer. A population-based case-control study in Sweden. Int J Cancer. 1994; 57: 26-31.

41. Inoue M, Tajima K, Hirose K, Kuroishi T, Gao CM, Kitoh T. Life-style and subsite of gastric cancer--joint effect of smoking and drinking habits. Int J Cancer. 1994; 56: 494-9.

42. D'Avanzo B, La Vecchia C, Franceschi S. Alcohol consumption and the risk of gastric cancer. Nutr Cancer. 1994; 22: 57-64. doi: 10.1080/01635589409514331.

43. Jedrychowski W, Boeing H, Wahrendorf J, Popiela T, Tobiasz-Adamczyk B, Kulig J. Vodka consumption, tobacco smoking and risk of gastric cancer in Poland. Int J Epidemiol. 1993; 22: 606-13.

44. Kabat GC, Ng SK, Wynder EL. Tobacco, alcohol intake, and diet in relation to adenocarcinoma of the esophagus and gastric cardia. Cancer Causes Control. 1993; 4: 123-32.

45. Hoshiyama Y, Sasaba T. A case-control study of stomach cancer and its relation to diet, cigarettes, and alcohol consumption in Saitama Prefecture, Japan. Cancer Causes Control. 1992; 3: 441-8.

46. Agudo A, Gonzalez CA, Marcos G, Sanz M, Saigi E, Verge J, Boleda M, Ortego J. Consumption of alcohol, coffee, and tobacco, and gastric cancer in Spain. Cancer Causes Control. 1992; 3: 137-43.

47. Choi SY, Kahyo H. Effect of cigarette smoking and alcohol consumption in the etiology of cancers of the digestive tract. Int J Cancer. 1991; 49: 381-6.

48. Tominaga K, Koyama Y, Sasagawa M, Hiroki M, Nagai M. A case-control study of stomach cancer and its genesis in relation to alcohol consumption, smoking, and familial cancer history. Jpn J Cancer Res. 1991; 82: 974-9.

49. Yu GP, Hsieh CC. Risk factors for stomach cancer: a population-based case-control study in Shanghai. Cancer Causes Control. 1991; 2: 169-74.

50. Boeing H, Frentzel-Beyme R, Berger M, Berndt V, Gores W, Korner M, Lohmeier R, Menarcher A, Mannl HF, Meinhardt M, et al. Case-control study on stomach cancer in Germany. Int J Cancer. 1991; 47: 858-64.

51. Wu-Williams AH, Yu MC, Mack TM. Life-style, workplace, and stomach cancer by subsite in young men of Los Angeles County. Cancer Res. 1990; 50: 2569-76.

52. Buiatti E, Palli D, Decarli A, Amadori D, Avellini C, Bianchi S, Bonaguri C, Cipriani F, Cocco P, Giacosa A, et al. A case-control study of gastric cancer and diet in Italy: II. Association with nutrients. Int J Cancer. 1990; 45: 896-901.

53. Kato I, Tominaga S, Ito Y, Kobayashi S, Yoshii Y, Matsuura A, Kameya A, Kano T. A comparative case-control analysis of stomach cancer and atrophic gastritis. Cancer Res. 1990; 50: 6559-64.

54. Hu JF, Zhang SF, Jia EM, Wang QQ, Liu SD, Liu YY, Wu YP, Cheng YT. Diet and cancer of the stomach: a case-control study in China. Int J Cancer. 1988; 41: 331-5.

55. You WC, Blot WJ, Chang YS, Ershow AG, Yang ZT, An Q, Henderson B, Xu GW, Fraumeni JF, Jr., Wang TG. Diet and high risk of stomach cancer in Shandong, China. Cancer Res. 1988; 48: 3518-23.

56. Correa P, Fontham E, Pickle LW, Chen V, Lin YP, Haenszel W. Dietary determinants of gastric cancer in south Louisiana inhabitants. J Natl Cancer Inst. 1985; 75: 645-54.

57. Tuyns AJ, Pequignot G, Gignoux M, Valla A. Cancers of the digestive tract, alcohol and tobacco. Int J Cancer. 1982; 30: 9-11.

58. Hoey J, Montvernay C, Lambert R. Wine and tobacco: risk factors for gastric cancer in France. Am J Epidemiol. 1981; 113: 668-74.

59. Ma SH, Jung W, Weiderpass E, Jang J, Hwang Y, Ahn C, Ko KP, Chang SH, Shin HR, Yoo KY, Park SK. Impact of alcohol drinking on gastric cancer development according to Helicobacter pylori infection status. Br J Cancer. 2015; 113: 1381-8. doi: 10.1038/bjc.2015.333.

60. Jayalekshmi PA, Hassani S, Nandakumar A, Koriyama C, Sebastian P, Akiba S. Gastric cancer risk in relation to tobacco use and alcohol drinking in Kerala, India--Karunagappally cohort study. World J Gastroenterol. 2015; 21: 12676-85. doi: 10.3748/wjg.v21.i44.12676.

61. Everatt R, Tamosiunas A, Kuzmickiene I, Virviciute D, Radisauskas R, Reklaitiene R, Milinaviciene E. Alcohol consumption and risk of gastric cancer: a cohort study of men in Kaunas, Lithuania, with up to 30 years follow-up. BMC Cancer. 2012; 12: 475. doi: 10.1186/1471-2407-12-475.

62. Duell EJ, Travier N, Lujan-Barroso L, Clavel-Chapelon F, Boutron-Ruault MC, Morois S, Palli D, Krogh V, Panico S, Tumino R, Sacerdote C, Quiros JR, Sanchez-Cantalejo E, et al. Alcohol consumption and gastric cancer risk in the European Prospective Investigation into Cancer and Nutrition (EPIC) cohort. Am J Clin Nutr. 2011; 94: 1266-75. doi: 10.3945/ajcn.111.012351.

63. Moy KA, Fan Y, Wang R, Gao YT, Yu MC, Yuan JM. Alcohol and tobacco use in relation to gastric cancer: a prospective study of men in Shanghai, China. Cancer Epidemiol Biomarkers Prev. 2010; 19: 2287-97. doi: 10.1158/1055-9965.epi-10-0362.

64. Steevens J, Schouten LJ, Goldbohm RA, van den Brandt PA. Alcohol consumption, cigarette smoking and risk of subtypes of oesophageal and gastric cancer: a prospective cohort study. Gut. 2010; 59: 39-48. doi: 10.1136/gut.2009.191080.

65. Song HJ, Kim HJ, Choi NK, Hahn S, Cho YJ, Park BJ. Gender differences in gastric cancer incidence in elderly former drinkers. Alcohol. 2008; 42: 363-8. doi: 10.1016/j.alcohol.2008.04.005.

66. Sung NY, Choi KS, Park EC, Park K, Lee SY, Lee AK, Choi IJ, Jung KW, Won YJ, Shin HR. Smoking, alcohol and gastric cancer risk in Korean men: the National Health Insurance Corporation Study. Br J Cancer. 2007; 97: 700-4. doi: 10.1038/sj.bjc.6603893.

67. Freedman ND, Abnet CC, Leitzmann MF, Mouw T, Subar AF, Hollenbeck AR, Schatzkin A. A prospective study of tobacco, alcohol, and the risk of esophageal and gastric cancer subtypes. Am J Epidemiol. 2007; 165: 1424-33. doi: 10.1093/aje/kwm051.

68. Larsson SC, Giovannucci E, Wolk A. Alcoholic beverage consumption and gastric cancer risk: a prospective population-based study in women. Int J Cancer. 2007; 120: 373-7. doi: 10.1002/ijc.22204.

69. Sjodahl K, Lu Y, Nilsen TI, Ye W, Hveem K, Vatten L, Lagergren J. Smoking and alcohol drinking in relation to risk of gastric cancer: a population-based, prospective cohort study. Int J Cancer. 2007; 120: 128-32. doi: 10.1002/ijc.22157.

70. Barstad B, Sorensen TI, Tjonneland A, Johansen D, Becker U, Andersen IB, Gronbaek M. Intake of wine, beer and spirits and risk of gastric cancer. Eur J Cancer Prev. 2005; 14: 239-43.

71. Nakaya N, Tsubono Y, Kuriyama S, Hozawa A, Shimazu T, Kurashima K, Fukudo S, Shibuya D, Tsuji I. Alcohol consumption and the risk of cancer in Japanese men: the Miyagi cohort study. Eur J Cancer Prev. 2005; 14: 169-74.

72. Lindblad M, Rodriguez LA, Lagergren J. Body mass, tobacco and alcohol and risk of esophageal, gastric cardia, and gastric non-cardia adenocarcinoma among men and women in a nested case-control study. Cancer Causes Control. 2005; 16: 285-94. doi: 10.1007/s10552-004-3485-7.

73. Sasazuki S, Sasaki S, Tsugane S. Cigarette smoking, alcohol consumption and subsequent gastric cancer risk by subsite and histologic type. Int J Cancer. 2002; 101: 560-6. doi: 10.1002/ijc.10649.

74. Galanis DJ, Kolonel LN, Lee J, Nomura A. Intakes of selected foods and beverages and the incidence of gastric cancer among the Japanese residents of Hawaii: a prospective study. Int J Epidemiol. 1998; 27: 173-80.

75. Nomura A, Grove JS, Stemmermann GN, Severson RK. A prospective study of stomach cancer and its relation to diet, cigarettes, and alcohol consumption. Cancer Res. 1990; 50: 627-31.
