# Supplementary material for: Alcohol drinking and gastric cancer risk: a meta-analysis of observational studies
Source: Oncotarget. 2017 Sep 15;8(58):99013–23. doi: 10.18632/oncotarget.20918 (PMC5716786; doi:10.18632/oncotarget.20918)
Supplement: Supplementary file 3 [file oncotarget-08-99013-s003.docx]

**Supplementary Table 2: Quality assessment of included studies according to the Newcastle-Ottawa scale**

Case-control studies

| Study | Year | Country | Selection | Comparability | Exposure | Total score |
| --- | --- | --- | --- | --- | --- | --- |
| Menezes | 2015 | Brazil | 2 | 1 | 2 | 5 |
| Hidaka | 2015 | Japan | 2 | 2 | 3 | 7 |
| Song | 2014 | China | 2 | 2 | 1 | 5 |
| Sun | 2013 | China | 3 | 2 | 1 | 6 |
| Matsuo | 2013 | Japan | 3 | 2 | 2 | 7 |
| Mao | 2011 | China | 3 | 1 | 1 | 5 |
| Gao | 2011 | China | 3 | 2 | 2 | 7 |
| Shin | 2011 | Korea | 2 | 2 | 1 | 5 |
| Wen | 2010 | China | 3 | 2 | 1 | 6 |
| Jorge | 2010 | Brazil | 3 | 1 | 1 | 5 |
| Nguyen | 2010 | Vietnam | 3 | 2 | 1 | 6 |
| Benedetti | 2009 | Canada | 3 | 2 | 2 | 7 |
| Lucenteforte | 2008 | Italy | 3 | 2 | 2 | 7 |
| Suwanrungruang | 2008 | Thailand | 3 | 1 | 1 | 5 |
| Boccia | 2007 | Italy | 3 | 2 | 1 | 6 |
| Fei&Xiao | 2006 | China | 3 | 1 | 1 | 5 |
| Lacasana-Navarro | 2006 | Mexico | 3 | 2 | 2 | 7 |
| Shen | 2004 | China | 3 | 2 | 1 | 6 |
| Nomura | 2003 | USA | 4 | 1 | 1 | 6 |
| Hamada | 2002 | Brazil | 2 | 2 | 2 | 6 |
| Nishimoto | 2002 | Brazil | 3 | 2 | 3 | 8 |
| Kikuchi | 2002 | Japan | 2 | 2 | 1 | 5 |
| Rao | 2002 | India | 2 | 1 | 2 | 5 |
| Wu | 2001 | USA | 4 | 2 | 1 | 7 |
| Munoz | 2001 | Venezuela | 2 | 2 | 1 | 5 |
| Chen | 2000 | China | 2 | 2 | 2 | 6 |
| Zaridze | 2000 | Russia | 3 | 2 | 2 | 7 |
| Mathew | 2000 | India | 3 | 2 | 2 | 7 |
| Lagergren | 2000 | Sweden | 4 | 2 | 2 | 8 |
| Ye | 1999 | Sweden | 4 | 2 | 2 | 8 |
| Chow | 1999 | Poland | 4 | 2 | 1 | 7 |
| Lopez-Carrillo | 1998 | Mexico | 4 | 2 | 1 | 7 |
| De Stefani | 1998 | Uruguay | 2 | 2 | 2 | 6 |
| Gammon | 1997 | USA | 3 | 2 | 2 | 7 |
| Gajalakshmi&Shanta | 1996 | India | 3 | 2 | 2 | 7 |
| Zhang | 1996 | USA | 3 | 2 | 2 | 7 |
| Ji | 1996 | China | 4 | 2 | 1 | 7 |
| Lee | 1995 | Korea | 3 | 2 | 1 | 6 |
| Falcao | 1994 | Portugal | 2 | 2 | 1 | 5 |
| Hansson | 1994 | Sweden | 3 | 2 | 2 | 7 |
| Inoue | 1994 | Japan | 3 | 2 | 2 | 7 |
| D’Avanzo | 1994 | Italy | 3 | 1 | 1 | 5 |
| Jedrychowski | 1993 | Poland | 2 | 2 | 1 | 5 |
| Kabat | 1993 | USA | 2 | 2 | 1 | 5 |
| Hoshiyama &Sasaba | 1992 | Japan | 4 | 2 | 1 | 7 |
| Agudo | 1992 | Spain | 2 | 2 | 1 | 5 |
| Choi & Kahyo | 1991 | Korea | 3 | 2 | 2 | 7 |
| Tominaga | 1991 | Japan | 3 | 0 | 2 | 5 |
| Yu&Hsieh | 1991 | China | 3 | 2 | 2 | 7 |
| Boeing | 1991 | Germany | 3 | 2 | 2 | 7 |
| Wu-Williams | 1990 | USA | 3 | 2 | 1 | 6 |
| Buiatti | 1990 | Italy | 3 | 2 | 2 | 7 |
| Kato | 1990 | Japan | 3 | 2 | 2 | 7 |
| Hu | 1988 | China | 3 | 2 | 1 | 6 |
| You | 1988 | China | 2 | 2 | 1 | 5 |
| Correa | 1985 | USA | 3 | 2 | 1 | 6 |
| Tuyns | 1982 | France | 1 | 2 | 1 | 4 |
| Hoey | 1981 | France | 2 | 2 | 1 | 5 |
| Average | | | 2.83 | 1.83 | 1.5 | 6.16 |

Cohort studies

| Study | Year | Country | Selection | Comparability | Outcome | Total score |
| --- | --- | --- | --- | --- | --- | --- |
| Ma | 2015 | Korea | 3 | 1 | 3 | 7 |
| Jayalekshmi | 2015 | India | 4 | 1 | 2 | 7 |
| Everatt | 2012 | Lithuania | 2 | 2 | 3 | 7 |
| Duell | 2011 | Europe | 3 | 2 | 3 | 8 |
| Moy | 2010 | China | 3 | 2 | 3 | 8 |
| Steevens | 2010 | Netherland | 2 | 2 | 3 | 7 |
| Song | 2008 | Korea | 3 | 1 | 3 | 7 |
| Sung | 2007 | Korea | 3 | 2 | 3 | 8 |
| Freedman | 2007 | USA | 3 | 2 | 2 | 7 |
| Larsson | 2006 | Sweden | 3 | 2 | 3 | 8 |
| Sjodahl | 2006 | Norway | 4 | 1 | 3 | 8 |
| Barstad | 2005 | Denmark | 4 | 2 | 3 | 9 |
| Nakaya | 2005 | Japan | 3 | 2 | 3 | 8 |
| Lindblad | 2005 | UK | 3 | 2 | 2 | 7 |
| Sasazuki | 2002 | Japan | 3 | 2 | 3 | 8 |
| Galanis | 1998 | USA | 2 | 2 | 3 | 7 |
| Nomura | 1990 | USA | 3 | 2 | 3 | 8 |
| Average | | | 3 | 1.76 | 2.83 | 7.59 |
